# Emerging roles of silicon in plant signaling networks and microbiome dynamics under salt stress

**DOI:** 10.3389/fpls.2026.1847453

**Published:** 2026-08-05

**Authors:** Jiuhong Chen, Yongfeng Li, Liqin Tuo

**Affiliations:** 1 Xinjiang Production and Construction Corps Second Division Agricultural Science Research Institute, Tiemenguan, Xinjiang, China; 2 College of Agriculture, Shihezi University, Shihezi, Xinjiang, China

**Keywords:** plant hormones, rhizosphere microbiota, salt stress, signal transduction, silicon

## Abstract

Silicon (Si) is widely recognized as a beneficial element that can improve plant performance under salt stress. However, a comprehensive understanding of its biological functions requires moving beyond isolated physiological responses toward an integrated view of plant signaling and rhizosphere processes. This narrative review critically synthesizes current evidence and proposes an “inside–outside” framework for Si-associated salt-stress mitigation. Internally, Si treatment has been reported to influence phytohormone homeostasis, particularly abscisic acid, jasmonic acid, and salicylic acid, while also affecting Ca^2+^-, nitric oxide-, and reactive oxygen species-related processes. These changes are associated with the regulation of stomatal behavior, root water transport, ion homeostasis, osmotic adjustment, antioxidant defense, and stress-responsive gene expression. Rather than acting as a universally established signal integrator, Si may modify the operating state, magnitude, and recovery kinetics of pre-existing stress-response networks by stabilizing membranes, restricting excessive Na^+^ accumulation, preserving K^+^ retention, and buffering cellular redox conditions. Externally, Si application can alter rhizosphere physicochemical properties, root-associated metabolites, and microbial community assembly. Si-associated enrichment of plant-beneficial microorganisms may contribute to nutrient cycling, ionic and osmotic regulation, redox protection, and plant growth, while microbial metabolites may reciprocally influence plant signaling and metabolism. Nevertheless, most microbiome functions remain inferred from community profiles and correlations, and causal validation is currently limited to a small number of experimental systems. Si uptake, transport, and spatial deposition provide the physiological basis for these interconnected responses, but their magnitude depends on plant species, genotype, Si-accumulation capacity, formulation, dose, application route, and stress intensity. This integrated framework identifies Si as a context-dependent modulator of plant–rhizosphere interactions and provides a mechanistic basis for developing precise and sustainable Si-based salinity-management strategies.

## 1 Introduction

Soil salinization is one of the most severe abiotic stresses constraining global agricultural sustainability. Currently, more than 20% of irrigated agricultural land worldwide is affected by salinization, and this proportion continues to increase as a result of climate change, sea-level rise, and inappropriate irrigation practices (Singh, 2021). Salt stress severely inhibits seed germination, plant growth and development, and final crop yield by inducing osmotic stress, ionic toxicity, nutritional imbalance, and secondary oxidative damage, thereby posing a substantial threat to global food security (Li et al., 2026; Qiao et al., 2026; Song et al., 2026; Wang et al., 2026a; Zhou et al., 2026). Consequently, the development of economical, efficient, and environmentally sustainable strategies to ameliorate saline soils and enhance crop salt tolerance has become an urgent priority in agricultural science and biotechnology.

Si, the second most abundant element in the Earth’s crust, has long been regarded primarily as a passive structural component in plants. Early research focused on its deposition in plant cell walls, where Si forms physical barriers that enhance mechanical strength, reduce transpiration, and protect against pests and diseases (Cai et al., 2009; Sun et al., 2010; Cao et al., 2016; Radhakrishnan et al., 2025; Pang et al., 2026). However, studies over the past two decades have substantially revised this conventional view. Accumulating evidence indicates that Si is not only actively taken up and translocated by plants via specific transporters, such as members of the Lsi family, but also exerts physiological, biochemical, and molecular regulatory functions in plant responses to a wide range of biotic and abiotic stresses (Moukhtari et al., 2026; Pang et al., 2026; Salam et al., 2026; Yucedag and Gailing, 2026). Under salt stress, Si application has been shown to significantly improve plant growth and physiological performance (Khalofah, 2026; Moukhtari et al., 2026; Wang et al., 2026d). This evolving understanding has shifted the perception of Si from a primarily physical enhancer to a multifunctional beneficial element involved in complex biological processes, thereby redirecting research efforts from phenomenological descriptions toward mechanistic investigation.

Although numerous reviews have summarized the physiological effects of Si in alleviating salt stress (Ma, 2004; Khan et al., 2019; Liu et al., 2019; Zhu et al., 2019b; Al Murad et al., 2020; Dhiman et al., 2021; Bayanati et al., 2023; Dabravolski and Isayenkov, 2024), the present review advances two key aspects that distinguish it from earlier syntheses. First, rather than describing individual Si effects in isolation, we propose an integrated dual-mode framework in which Si simultaneously functions as an internal signaling network integrator, modulating Ca^2+^/NO second messengers and ABA/JA/SA hormonal crosstalk, and as an external rhizosphere ecosystem engineer. This “inside-outside” coupling represents a conceptual shift from previous reviews that have largely focused on either physiological or molecular mechanisms separately. Second, we elevate the microbiome dimension from a supplementary topic to a core mechanistic component, synthesizing emerging evidence on how Si shapes microbial community assembly and how this, in turn, feeds back into plant signaling. By synthesizing evidence from molecular signaling to ecological interactions, this review aims to establish a coherent theoretical framework for understanding Si’s biological functions and for informing Si-based agricultural strategies.

## 2 Si uptake, distribution, and the regulation of its homeostasis

### 2.1 Key transporters for Si uptake and long-distance transport (Lsi family) and their expression regulation under salt stress

**Figure 1:**
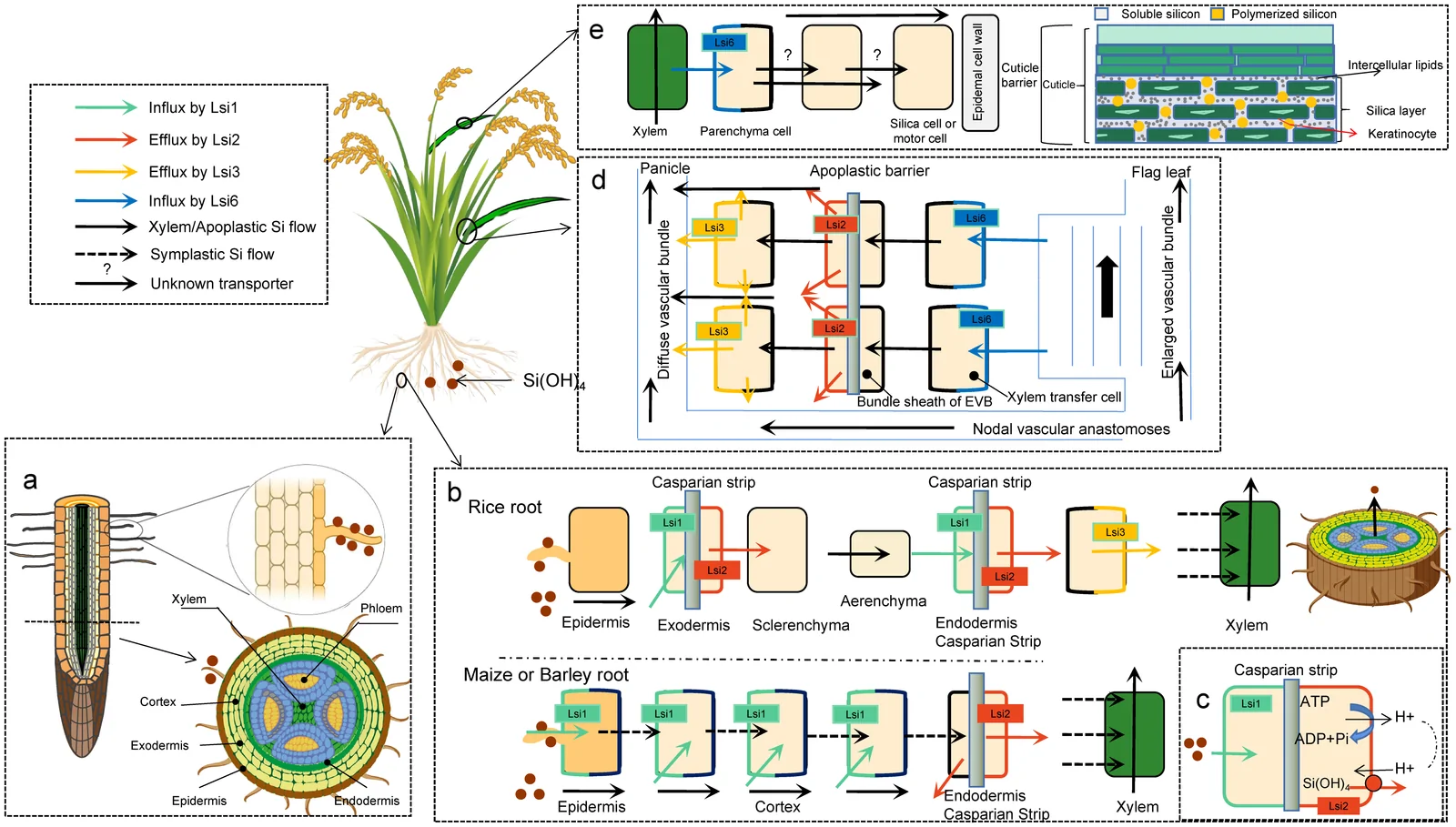
A schematic model of Si uptake and transport in plants. **(A)** Schematic diagram of longitudinal and cross-section of the root system. **(B)** Generalized pathway showing Si uptake and transport via transporters in Rice, Maize and Barley. **(C)** Lsi1 and Lsi2 are localized to the plasma membrane of both exodermis and endodermis. Lsi1 mediates the thermos dynamically passive uniport of Si(OH)_4_, whereas Lsi2 is thought to mediate the secondary active transport of Si(OH)_4_ in antiport with H^+^ (the electrochemical gradient of which is generated by the plasma membrane H^+^-ATPase). **(D)** Schematic presentation of intervascular transfer pathway of Si in node. Si in the xylem of an enlarged vascular bundle is unloaded by Lsi6 polarly localized at the Xylem transfer cells. **(E)** possible Si unloading and deposition pathway in the leaf, Si precipitates in the cuticle of the leaf to form a Si layer.

Efficient uptake and internal distribution of Si in plants depend on a set of highly specific transporter proteins, among which the Lsi family plays a central role. Under salinity stress, fine-tuned regulation of these transporters constitutes an early molecular event by which plants adjust Si homeostasis and initiate defense responses. Silicic acid (Si(OH)_4_) traverses the root cortex, crosses the Casparian strip, enters the xylem, and is ultimately translocated to epidermal tissues of shoots and leaves, where it accumulates (**Figure 1**) (Ma and Yamaji, 2006). Multiple transporters and aquaporins have been identified as mediators of Si uptake and translocation from roots to shoots (**Table 1**). Through coordinated activity and distinct polar localization, the transporters Lsi1 and Lsi2 facilitate directional Si transport from roots to aerial tissues (Ma and Yamaji, 2006). Lsi1 functions as a Si influx transporter localized on the distal side of the plasma membrane of root exodermal and endodermal cells, mediating the uptake of silicic acid from the soil solution into root tissues (Wu and Beitz, 2007). In contrast, Lsi2 functions as an efflux transporter localized on the proximal side of the plasma membrane in root exodermal and endodermal cells, exporting intracellular silicic acid into the root apoplast for subsequent xylem loading (**Figures 1A–C**) (Ma et al., 2007). Recent studies further demonstrate that OsLsi3 is required for efficient Si transfer from rice roots to shoots (**Figure 1B**) (Huang et al., 2022). Homologs of Lsi1 and Lsi2 have been identified in multiple plant species, including pumpkin (Mitani et al., 2011; Mitani-Ueno et al., 2011), pepper (Gómez-Merino et al., 2020), sorghum (Soukup et al., 2017), maize (Bokor et al., 2015), horsetail (Vivancos et al., 2016), and rice (Ma et al., 2007). However, their cellular localization and polarity vary among species, particularly within the Poaceae family (Mitani et al., 2009; Mitani-Ueno and Ma, 2021). Inter-species variation in Si accumulation is largely attributable to differences in transporter presence, expression levels, tissue localization, and membrane polarity (Mitani-Ueno and Ma, 2021). Driven by transpiration flow, Si is transported via Lsi6 from the xylem into shoot parenchyma cells (Ma et al., 2011; Pontigo et al., 2015). Notably, certain tissues, such as rice nodes, accumulate exceptionally high levels of Si despite relatively low transpiration rates (**Figure 1D**). Preferential Si distribution in rice depends on intervascular transfer mediated by Lsi2, Lsi3, and Lsi6. Lsi3 is localized between diffuse and enlarged vascular bundles in parenchyma tissues, whereas Lsi2 exhibits polar localization in the bundle sheath cells surrounding large vascular bundles, adjacent to the xylem transfer cells expressing Lsi6 (Yamaji et al., 2015). Loss-of-function mutants of OsLsi2 and OsLsi3 display phenotypes similar to OsLsi6 mutants, showing increased Si allocation to flag leaves and reduced distribution to panicles. Ultimately, Si distribution and deposition in shoot and leaf epidermal cells are determined by Lsi6-mediated transport of Si(OH)_4_ from the xylem into xylem parenchyma cells (**Figure 1E**). Within these cells, Si is deposited as polymerized silica (SiO_2_·nH_2_O), either within phyllosilicate structures or as a gel beneath the cuticle, forming a bilayered cuticular structure.

**Table 1:** Si specific genes and transporters or sub families aquaporins of various plants.

Transporter/aquaporin	Plant species	Specific genes	Type and expression sites	Reference(s)
Lsi1	Rice (*Oryza sativa*)	*OsLsi1*	Influx; roots	(Ma et al., 2006)
	Barley (*Hordeum vulgare* L.)	*HvLsi1*	Influx; roots	(Chiba et al., 2009)
	Wheat (*Triticum aestivum* L.)	*TaLsi1*	Influx; roots	(Montpetit et al., 2012)
	Tomato (*Solanum lycopersicum* L.)	*SlLsi1*	Influx; roots	(Sun et al., 2020)
	Maize (*Zea mays* L.)	*ZmLsi1*	Influx; roots	(Mitani et al., 2008)
	Sorghum (*Sorghum bicolor*)	*SbLsi1*	Influx; roots and leaves	(Markovich et al., 2019, 2022)
	Potato (*Solanum tuberosum*)	*StLsi1*	Influx; roots and leaves	(Vulavala et al., 2016)
	Pepper (*Capsicum annuum*)	*CaLsi1*	Influx; roots	(Gómez-Merino et al., 2020)
	Poplar (*Populus trichocarpa*)	*PtLsi1*	Influx; roots and shoots	(Hassan et al., 2022)
	Pumpkin (*Cucurbita moschata*)	*CmLsi1*	Influx; roots and shoots	(Mitani-Ueno et al., 2011)
	Cucumber (*Cucumis sativus*)	*CsLsi1*	Influx; roots	(Sun et al., 2017)
	Tobacco (*Nicotiana sylvestris*)	*NsLsi1*	Influx; roots and shoots	(Coskun et al., 2019)
Lsi2	Rice (*Oryza sativa*)	*OsLsi2*	Efflux; roots	(Mitani-Ueno et al., 2016)
	Barley (*Hordeum vulgare* L.)	*HvLsi2*	Efflux; roots	(Mitani et al., 2009)
	Maize (*Zea mays* L.)	*ZmLsi2*	Efflux; roots	(Mitani et al., 2009)
	Pumpkin (*Cucurbita moschata*)	*CmLsi2-1* *CmLsi2-2*	Efflux; roots and shoots	(Ma et al., 2011)
	Cucumber (*Cucumis sativus*)	*CsLsi2*	Efflux; roots	(Sun et al., 2018)
	Pepper (*Capsicum annuum*)	*CaLsi2*	Efflux; roots	(Gómez-Merino et al., 2020)
	Horsetail (*Equisetum arvense*)	*EaLsi2–1* EaLsi2-2	Efflux; roots and shoots	(Vivancos et al., 2016)
	Poplar (*Populus trichocarpa*)	*PtLsi2*	Efflux; roots and shoots	(Hassan et al., 2022)
Lsi3	Rice (*Oryza sativa*)	*OsLsi3*	Efflux; roots	(Huang et al., 2022)
Lsi6	Rice (*Oryza sativa*)	*OsLsi6*	Influx; leaf sheath, leaf blades, nodes and root tips	(Yamaji et al., 2008; Yamaji and Ma, 2009)
	Barly (*Hordeum vulgare*)	HvLsi6	Influx; roots and shoots	(Yamaji et al., 2012)
	Maize (*Zea mays* L.)	*ZmLsi6*	Influx; leaf sheath and blades	(Mitani et al., 2008)
Some other families of aquaporins like MIP, NIP etc.	horsetail (*Equisetum arvense*)	*EaNIP3;1 EaNIP3;3*	Influx; roots and shoots	(Grégoire et al., 2012)
	Date palm (*Phoenix dactylifera*)	*PdNIP2*	Influx; roots	(Bokor et al., 2019)
	Soybean (*Glycine max*)	*GmNIP2–1 GmNIP2-2*	Influx; roots and shoots	(Deshmukh et al., 2013)
	Grapevine (*Vitis vinifera* L.)	*VvNIP2;1*	Influx; roots and green berries	(Noronha et al., 2020)
	Tobacco (*Nicotiana benthamiana*)	*NtNIP2;1*	Influx; roots	(Zellner et al., 2019)
	Sorghum (*Sorghum bicolor*)	*SbPIP1;6* *SbPIP2;2* *SbPIP2;6*	Influx; roots	(Liu et al., 2015)

In several plant species, Si supplementation under salinity has been associated with altered or increased expression of Si transporter genes. Studies on grapevine genotypes with contrasting salt tolerance showed that Si application upregulates the expression of the transporter genes *VvNIP2;1* and *VvLsi2* (Heidarpour et al., 2025). Under salt stress, Si nanoparticles were reported to enhance the expression of several aquaporin genes (*CsPIP1;1*, *CsPIP2;3*, and *CsTIP4;1*) in ‘Valencia’ sweet orange (Mahmoud et al., 2022). Similarly, Si treatment induced the upregulation of Si transporter genes (*Lsi1*, *Lsi2*, and *Lsi3*) in salt-stressed mung bean (Al Murad and Muneer, 2023). Collectively, these findings suggest that plants enhance their Si uptake and transport capacity under salt stress, thereby contributing to stress mitigation. The functional significance of Si transporters under salinity has been further validated through overexpression studies and loss-of-function mutants. Heterologous expression of the sugarcane Si transporter gene *SspNIP2* in tobacco produced transgenic lines with improved growth performance compared with wild-type plants under salt stress (Park et al., 2015). In rice, Si application promoted growth and reduced Na^+^ translocation to root tips in wild-type plants under salinity, but not in *lsi1* and *lsi2* mutants. Further analysis showed Si deposition in the root endodermis of wild-type plants, which was absent in the corresponding mutants (Yan et al., 2021). Collectively, these results indicate that salt stress–associated Si accumulation depends on the expression and function of Lsi-family transporters. However, the upstream signaling pathways linking salt stress perception to the transcriptional regulation of Lsi transporter genes remain largely unresolved.

Despite the central role of Lsi-family transporters in Si uptake and long-distance transport, several physiological and agronomic constraints should be considered when interpreting transporter-based Si homeostasis under salt stress. First, Si uptake is transporter-mediated but not unlimited. Classical kinetic analysis in rice roots showed that Si uptake follows a saturable process, with an estimated Km of approximately 0.32 mM, indicating that increased external Si supply does not necessarily lead to proportional increases in Si uptake once transporter capacity becomes limiting (Tamai and Ma, 2003). In addition, shoot Si status can feed back on root Si acquisition. Mitani-Ueno et al. (2016) showed that Si accumulation in shoots is required for the downregulation of OsLsi1 and OsLsi2 expression in rice roots, while Yamaji et al. (2024) further identified a shoot-derived signaling protein involved in regulating root Si uptake. Therefore, salt-induced changes in Lsi expression should be interpreted as part of a regulated Si homeostasis system rather than as evidence that plants can continuously enhance Si uptake without physiological limits.

Second, the energetic basis of Si transport remains incompletely understood. Lsi1 is an aquaporin-like influx channel that facilitates the movement of uncharged silicic acid, whereas Lsi2 is generally proposed to mediate Si efflux and xylem loading, potentially through a proton-coupled mechanism. However, the structure–function relationship of Lsi2 and the direct evidence for a Si(OH)_4_/H^+^ antiport mechanism remain insufficiently resolved (Coskun et al., 2021). This uncertainty is important under salt stress because plants already invest substantial energy in maintaining ion homeostasis, osmotic adjustment, membrane potential and H^+^-ATPase-dependent electrochemical gradients. Thus, Si transport under salinity may involve indirect physiological costs associated with maintaining membrane gradients, transporter polarity and root-zone Si mobilization, particularly when plant-available Si is low.

Third, most mechanistic evidence for Lsi-mediated Si uptake has been generated under hydroponic, nutrient-solution, mutant or heterologous expression systems, where Si concentration, pH, ionic composition and salinity are tightly controlled. These systems are essential for identifying transporter function, but they do not fully represent field soils. In soil, plant-available Si varies with parent material, weathering intensity, clay minerals, Fe/Al oxides, pH, organic matter, salinity, irrigation regime and fertilizer source. Large-scale soil studies show that plant-available Si is shaped by soil properties and agricultural practices, even though total Si is abundant in soils (Caubet et al., 2020). Similarly, sugarcane studies demonstrated that the total Si content of fertilizer sources does not necessarily predict plant-available soil Si or leaf Si accumulation, because source mineralogy, solubility and soil pH correction strongly influence Si release and uptake (Keeping, 2017).

These soil-dependent constraints have important implications for the field relevance of Lsi-mediated Si uptake under salt stress. In hydroponics, transporter induction can be directly linked to external silicic acid supply and plant Si accumulation. In field soils, however, increased Lsi expression may not translate into greater Si acquisition if soluble Si is limited, if Si is adsorbed or polymerized, if salinity restricts root activity, or if transpiration-driven xylem transport is reduced. Moreover, Si supply can interact with the uptake and mobility of other nutrients, including phosphorus and several essential or beneficial elements, through rhizosphere and plant-mediated processes (Pavlovic et al., 2021). Therefore, Lsi-family transporters should be viewed not only as molecular determinants of Si accumulation, but also as components of a broader soil–plant regulatory system whose function depends on transporter kinetics, feedback regulation, root-zone chemistry, plant energetic status and field-scale Si bioavailability.

Taken together, current evidence supports a conditional model in which salt stress may modulate Si transporter expression and thereby contribute to Si-mediated stress mitigation. However, the magnitude of this response depends on transporter saturation, shoot-derived feedback regulation, the energetic and electrochemical context of root transport, and the availability of soluble Si in the rhizosphere. Future studies should integrate hydroponic and soil-based experiments, quantify Lsi transporter kinetics under saline conditions, compare genotypes across contrasting soil Si backgrounds, and test whether stress-induced Lsi expression reliably predicts Si accumulation, yield protection and salt tolerance under multi-season field conditions.

### 2.2 Spatial mapping of defense functions: strategic deployment and precise positioning

Si distribution within plants exhibits pronounced tissue- and cell-type specificity. This heterogeneous deposition pattern provides the spatial basis for its defensive functions. Si primarily accumulates in the cell walls of the root epidermis, exodermis, and endodermis, forming a dual barrier that regulates Si uptake and restricts the entry of harmful ions such as Na^+^ (Lux et al., 2003; Yang et al., 2024; Sogarwal et al., 2025). Endodermal deposition is particularly important, as it reinforces the Casparian strip and modulates apoplastic transport (Lux et al., 2003; Zexer et al., 2024). Si deposition in xylem vessel walls and bundle sheath cells further enhances the mechanical strength of conduits involved in water and silicic acid transport (Goto et al., 2003; Yan et al., 2020). The highest Si concentrations are typically observed in leaves, particularly within epidermal cells, silica cells (silica bodies), and cell walls (Javaid et al., 2019; Avestan et al., 2021).

Si interacts with cell wall components, including cellulose, hemicellulose, pectin, and lignin, through covalent or hydrogen bonding, forming Si–cellulose–lignin complexes (Williams, 2007; He et al., 2015; Cui et al., 2020; Sheng and Chen, 2020; Radotić et al., 2022). These complexes enhance cell wall mechanical strength and rigidity (Liang et al., 2005), and reduce Na^+^ influx through the apoplastic pathway (Haque and Matsubara, 2018). These complexes may also influence the activity of cell wall–associated sensing proteins. Additionally, Si deposition associated with the plasma membrane can alter lipid composition, membrane fluidity, and microdomain organization, thereby stabilizing the membrane under salt stress and potentially modulating the function of membrane-anchored receptor proteins (Samuels et al., 1994; Jiang et al., 2023). Si has also been reported to exhibit preferential distribution near organelles involved in stress responses, such as chloroplasts and vacuoles (Vaculík et al., 2015; Bari et al., 2020; Ju et al., 2020; Martín-Esquinas and Hernández-Apaolaza, 2021). For instance, Si deposition in mesophyll cells could provide physical shielding for chloroplasts, mitigating photoinhibition (Cai et al., 2025). Overall, the spatially resolved deposition of Si at cell walls and specific interfaces not only establishes a static physical barrier against salt ion intrusion and oxidative damage, but also creates a unique microenvironment capable of modulating water transport, ion fluxes, and even cellular signal perception.

### 2.3 Temporal remodeling of ionic and redox homeostasis: early signaling and later stabilization

Salt stress disrupts ionic and redox homeostasis through temporally distinct but closely interconnected processes. Plant responses to salinity involve a rapid phase of stress perception and signal initiation, followed by slower processes of ion redistribution, metabolic acclimation, and growth adjustment (Ma et al., 2026). For the purpose of this review, early responses are operationally defined as events occurring from minutes to 24 h after salt exposure, including changes in membrane potential, transient Ca^2+^ and reactive oxygen species (ROS) signals, initial regulation of ion transporters, and activation of antioxidant networks. Later responses occur after 24 h and may extend over several days or weeks, encompassing cumulative Na+ exclusion and compartmentalization, K^+^ retention, osmolyte accumulation, sustained limitation of oxidative injury, and stabilization of photosynthesis and growth. These temporal boundaries are approximate because response kinetics vary with species, genotype, tissue, developmental stage, salt intensity, and experimental conditions. The transient nature of early ROS and Ca^2+^ signals and the pronounced spatial and temporal variation in K^+^/Na^+^ regulation further indicate that ionic and redox responses should not be interpreted from a single sampling point (Jiménez et al., 2026; Tanveer et al., 2026).

At the ionic level, the magnitude of Si-mediated regulation is substantial but strongly context dependent. Across representative direct comparisons in maize and olive, Si reduced measured Na^+^ endpoints by approximately 16% to 58%. This range includes total leaf Na^+^ concentration, apoplastic Na^+^ concentration, and Na^+^ accumulation in newly developed leaves and should therefore be interpreted as a descriptive comparative range rather than a pooled effect estimate. In maize exposed to 100 mM NaCl, Ca_2_SiO_4_ supplementation reduced total leaf Na^+^ concentration by approximately 16% to 28%, depending on genotype and leaf age. In younger leaves, apoplastic Na^+^ concentration was reduced by 33% to 48%, whereas K^+^ concentration in older leaves increased by 30% to 37% relative to salt treatment alone (Mahmood et al., 2024). In the salt-sensitive olive cultivar ‘Leccino’, Si priming reduced Na^+^ accumulation in newly developed leaves by 58% and maintained K^+^ concentration after 51 days of salinity, resulting in an improved K^+^/Na^+^ balance (Fidalgo-Illesca et al., 2025). In alfalfa exposed to 50 to 200 mM NaCl for 14 days, Si consistently reduced leaf Na^+^ accumulation and increased leaf K^+^ concentration. Estimates based on the treatment means presented by Meng et al. (2020) indicate that the K^+^/Na^+^ ratio increased by approximately 40% to 140%, depending on salinity intensity. Together, these comparisons demonstrate that Si effects on Na^+^ accumulation and the K^+^/Na^+^ balance vary considerably among species, genotypes, tissues, salinity levels, and treatment durations.

**Figure 2:**
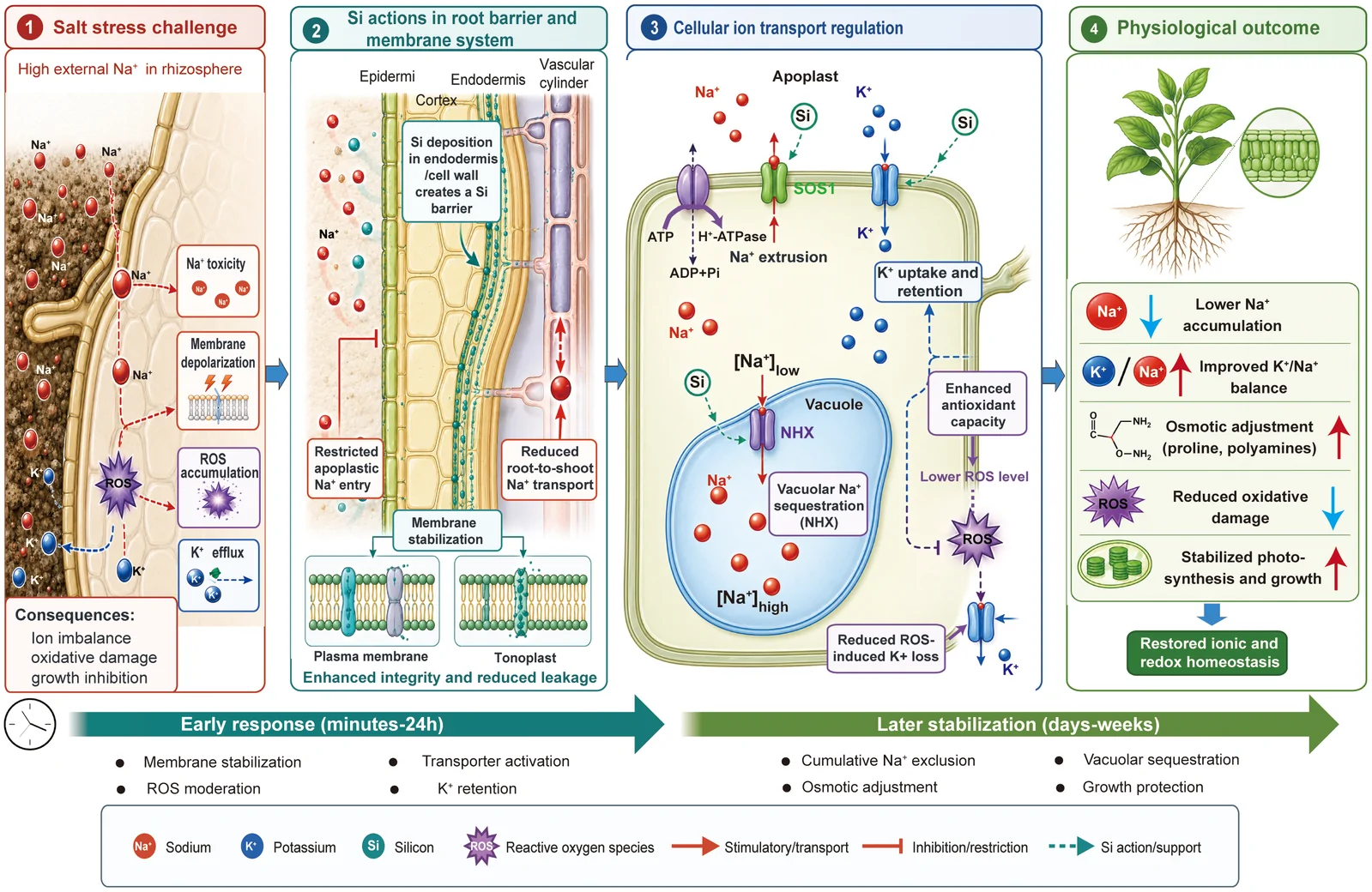
A proposed model of Si-associated regulation of ionic homeostasis under salt stress. Salt stress induces excessive Na^+^ accumulation, membrane depolarization, ROS overproduction, and K^+^ loss, resulting in ionic imbalance and cellular injury. Si alleviates ionic toxicity by reinforcing root apoplastic barriers, restricting Na^+^ entry and root-to-shoot translocation, and stabilizing plasma membrane and tonoplast integrity. At the cellular level, Si treatment has been associated with increased expression of SOS- and NHX-related genes, improved Na^+^ exclusion and compartmentalization, and enhanced K^+^ retention. Si-enhanced antioxidant capacity further limits ROS-induced K^+^ efflux and membrane damage. These coordinated processes maintain K^+^/Na^+^ homeostasis, reduce oxidative stress, and contribute to improved salinity tolerance. Early responses (minutes to 24 h) involve rapid regulation of ion transport and redox signaling, whereas later responses (days to weeks) establish sustained ionic balance, osmotic adjustment, and growth recovery.

Mechanistically, Si regulates ion homeostasis through several complementary processes. Si deposition in the root endodermis and cell walls strengthens apoplastic barriers and restricts passive Na^+^ entry and root-to-shoot transport (**Figure 2**) (Fleck et al., 2010; Yan et al., 2020, 2021). Si also contributes to the stabilization of the plasma membrane and tonoplast, thereby maintaining the electrochemical conditions required for ion transport (Gao et al., 2007; Heidarpour et al., 2025). In cucumber roots, Si reduced salt-induced and ROS-induced K^+^ efflux, enhanced root Na^+^ exclusion, increased antioxidant capacity, and upregulated genes associated with the SOS and NHX transport systems (Yan et al., 2025). These processes support cytosolic Na^+^ extrusion, vacuolar Na^+^ sequestration, and K^+^ retention (**Figure 2**). The resulting improvement in the K^+^/Na^+^ balance should therefore be understood as an integrated outcome of restricted Na^+^ influx and translocation, enhanced Na^+^ exclusion and compartmentalization, and protection of K^+^ uptake and retention, rather than as the consequence of a single transporter or physical barrier.

Si also promotes osmotic adjustment through the accumulation of compatible solutes, including proline and polyamines (Li et al., 2004; Yin et al., 2016; Zhu et al., 2020; Cui et al., 2021). Increased proline accumulation contributes to cellular water retention and macromolecular stabilization, whereas polyamines may influence membrane properties and ion-channel activity. Regulation of membrane transport, ion fluxes, and osmolyte metabolism may begin during the early response phase. However, changes in whole-tissue Na^+^ content, K^+^/Na^+^ ratios, water status, and biomass usually represent the cumulative outcome of these processes over several days or weeks. Early activation of ion-regulatory mechanisms should therefore not be equated with the immediate completion of whole-plant ion homeostasis.

Redox regulation exhibits a similarly dynamic and time-dependent pattern. Si should not be regarded simply as an antioxidant or as a treatment that continuously increases antioxidant enzyme activities. Instead, Si modulates the balance among ROS production, scavenging, and signaling according to stress intensity, tissue context, and response stage. During the early phase, Si may enhance or stabilize the activities of antioxidant enzymes, including superoxide dismutase (SOD), catalase (CAT), ascorbate peroxidase (APX), peroxidase (POD), and glutathione reductase (GR), while supporting non-enzymatic antioxidants and the ascorbate-glutathione cycle. These responses limit excessive ROS accumulation and lipid peroxidation while potentially preserving controlled ROS signals that participate in downstream stress responses (Jiménez et al., 2026). In rice exposed to 50 mM NaCl, salt stress increased MDA and H_2_O_2_ contents by 78% and 67%, respectively. Foliar application of Si oxide nanoparticles enhanced SOD, POD, and CAT activities and reduced the accumulation of these oxidative-damage markers (Alam et al., 2025). In cucumber roots, Si-enhanced antioxidant capacity was accompanied by reduced ROS-induced K^+^ efflux, demonstrating a direct functional connection between redox regulation and ion homeostasis (Yan et al., 2025).

The direction and magnitude of antioxidant responses also depend on sampling time. Time-resolved analysis of salinity tolerance showed that quinoa exhibited a transient increase in H_2_O_2_ followed by rapid recovery, whereas spinach displayed prolonged ROS accumulation and delayed antioxidant activation. In quinoa roots, intracellular K^+^ in the elongation zone initially decreased at 2 h but recovered by 24 h, illustrating that early ion loss and later homeostatic recovery may occur within the same tissue (Tanveer et al., 2026). Although this comparison did not involve Si treatment, it provides a mechanistic basis for interpreting Si-related antioxidant and ion data within an explicit temporal framework. An early increase in antioxidant activity may indicate rapid defense activation, whereas a later decrease may reflect reduced oxidative pressure rather than weakened protection. Consequently, later Si effects are more appropriately evaluated through sustained reductions in ROS, MDA, electrolyte leakage, and membrane injury, together with recovery of photosynthesis, biomass, and yield.

Collectively, current evidence suggests that Si treatment can influence early stress responses and contribute to the subsequent stabilization of ionic, osmotic, and redox homeostasis. By limiting excessive Na^+^ accumulation, preserving K^+^ retention, and preventing uncontrolled oxidative damage, this temporally coordinated response establishes an intracellular environment that supports the transmission and integration of hormonal signals and second messengers, including ABA, JA, Ca^2+^, and NO. Si therefore functions not only as a restorer of cellular homeostasis but also as a coordinator of the signaling networks discussed in the following section.

## 3 Core mechanism I: potential roles of Si in stress-signaling integration

### 3.1 Si-mediated modulation of hormonal responses under salt stress

Salt stress triggers a cascade of molecular responses in plants, among which the dynamic reprogramming of phytohormone signaling networks plays a central role. Increasing evidence suggests that Si functions far beyond the modulation of individual signaling pathways; rather, it acts as a coordinating component within integrated stress-responsive signaling systems. Available studies indicate that Si treatment can alter phytohormone abundance, hormone-related gene expression, and downstream physiological responses under salinity (Kim et al., 2014; Liu et al., 2024; Xiong et al., 2025; Zhao et al., 2025). This section critically examines current evidence on how Si treatment influences major phytohormone-related responses under salt stress conditions.

ABA serves as a central regulator of plant responses to salt stress. The interplay between Si and ABA extends beyond alterations in endogenous ABA accumulation and primarily involves enhancement of ABA biosynthesis and signal transduction (Kim et al., 2014; Zhu et al., 2019a; Wang et al., 2026d). The Na+Si treatment markedly induced the expression of the ABA biosynthetic genes *SlNCED1* and *SlNCED2* in tomato seedling leaves relative to the NaCl treatment (Wang et al., 2026d). In tobacco, exogenous Si similarly enhanced ABA content, an effect attributed to the upregulated expression of *NtNCED1* and *NtNCED5* (Liu et al., 2024). A significant increase in ABA content was observed following Si treatment, accompanied by improved root water uptake efficiency and enhanced leaf water status. To determine the involvement of ABA in Si-mediated root hydraulic conductance, sodium tungstate, an inhibitor of ABA biosynthesis, was employed. The promoting effect of Si on root hydraulic conductance was substantially diminished upon inhibition of ABA synthesis, indicating that ABA plays a critical mediatory role in Si-modulated root water transport (Liu et al., 2024). One of the most prominent physiological manifestations of this synergy is observed in stomatal regulation. Under salt stress, Si application enhances photosynthetic performance by modulating stomatal closure and reducing water loss (Fidalgo-Illesca et al., 2025). Nevertheless, the precise molecular mechanisms by which Si mediates ABA-dependent stomatal regulation have yet to be fully clarified. Under drought stress, the combined application of Bacillus pumilus G5 and Si elevated the enzymatic activities of Ctrz and NCED without altering endogenous ABA concentrations in drought-stressed plants, suggesting that this combined treatment is conducive to maintaining ABA homeostasis. The underlying mechanism likely involves accelerating the dynamic turnover between ABA biosynthesis and catabolism, thereby activating PYR/PYL receptors and SnRK2 kinases to enhance plant drought tolerance (Wang et al., 2026c). By contrast, the molecular mechanisms underlying Si-mediated ABA signaling under salt stress warrant further investigation. Moreover, Si does not consistently promote ABA accumulation under saline conditions. For instance, exogenous application of Si nanoparticles (SiNPs) under salt stress was found to upregulate the expression of the ABA catabolic gene CYP707A2 while suppressing the ABA biosynthetic genes NCED1 and NCED2 (Wang et al., 2024b). In the grapevine cultivar ‘Castellana Negra’, exposure to 90 mM NaCl for 75 days increased ABA content by 39% relative to the water control, whereas the addition of 10 mM Si maintained ABA at levels comparable to those of the control (Urbano-Gálvez et al., 2025). An earlier study reported that Si-treated rice exhibited significantly elevated ABA levels at 6 h and 12 h after treatment, but no differences were observed at 24 h; consistent with this, *NCED1* and *NCED4* were upregulated at 6 h and 12 h but downregulated at 24 h (Kim et al., 2014). Under 200 mM NaCl stress, the application of 0.3 mM Si upregulated the expression of *CYP707A1*, which encodes ABA 8′-hydroxylase, at 12 h after germination onset, whereas at 36 h post-germination, it significantly suppressed the expression of the ABA biosynthetic genes *NCED1* and *NCED2* (Gou et al., 2020). Collectively, these observations underscore the complexity of Si-regulated ABA accumulation under salt stress, which warrants further elucidation through integrated approaches encompassing temporal dynamics, multi-omics profiling, and regulatory network analyses.

JA has emerged as a key regulator of ROS homeostasis and signaling. Under salt stress, JA is converted to its bioactive conjugate JA-isoleucine (JA-Ile), which is subsequently imported into the nucleus by the transporter AtJAT1/AtABCG16 and perceived by the SCF^COI1–JAZ co-receptor complex (Wang et al., 2019). This perception triggers ubiquitination of JAZ proteins and their degradation via the 26S proteasome (Raza et al., 2021), thereby releasing MYC transcription factors to activate the transcription of genes involved in defense, stress responses, and JA biosynthesis (Song et al., 2022). The JA signaling pathway reprograms plant metabolism by driving the accumulation of antioxidants and secondary metabolites, including flavonoids, alkaloids, terpenes, and phenolics, thus modulating ROS production and scavenging (Sheteiwy et al., 2022). Earlier studies indicated that short-term Si application under salt stress led to a marked downregulation of JA, a hormone closely associated with stress and defense responses (Kim et al., 2014). Subsequent investigations in maize revealed that salt stress elevated endogenous JA levels, whereas Si supplementation significantly reduced them (Kubi et al., 2021). A similar maintenance of JA content by Si under salinity was also reported in grapevine (Urbano-Gálvez et al., 2025). Despite these observations, the Si-mediated regulation of JA biosynthesis and signal transduction under salt stress remains poorly understood. Under drought conditions, combined treatment with Bacillus pumilus G5 and Si promoted JA signaling and upregulated the expression of the transcription factor *MYC2*, effectively alleviating drought-induced damage (Wang et al., 2026c). ABA and JA function as interconnected molecular hubs within the hormonal crosstalk network, operating through shared transcription factors (MYC2, ABI5), relevant protein–protein interactions (JAZ–ABI5, JAZ–DELLA), and overlapping downstream effectors (Song et al., 2021; Han et al., 2023; Lamers et al., 2025). This intricate interplay enables plants to fine-tune the magnitude and duration of stress responses in a manner dependent on salt concentration and developmental stage. Notably, JA production is triggered later than ABA but exhibits comparable or even greater intensity under salt stress (Moons et al., 1997), suggesting that Si-alleviated salinity responses may be influenced by the temporal dynamics of ABA–JA signaling. Nevertheless, the molecular mechanisms, network modelling, and temporal signaling dynamics of ABA–JA crosstalk following exogenous Si application have not yet been documented.

SA is a naturally occurring phenolic molecule that serves as a pivotal component of the complex plant signaling network, regulating diverse physiological processes essential for environmental adaptation, growth, and development (Sharma et al., 2023). Under salt stress, exogenous SA application alleviates growth inhibition, enhances the activity of the antioxidant system, and reduces hydrogen peroxide accumulation, lipid peroxidation, and ion leakage (Kashif et al., 2024; Maslennikova et al., 2024). Early studies established that SA-mediated pathways participate in cellular ROS scavenging and promote antioxidant metabolism (Overmyer et al., 2003). In rice exposed to 100 mM NaCl, short-term (6 h) treatment with 0.5 mM sodium metasilicate led to a marked increase in SA content, whereas no significant change was observed at 12–24 h post-application (Kim et al., 2014). A more recent investigation on grapevine ‘Castellana Negra’ revealed that, after 75 days of treatment with 90 mM NaCl and 10 mM calcium silicate, Si supplementation reduced SA levels relative to those under salt stress alone (Urbano-Gálvez et al., 2025). These findings suggest that Si contributes to maintaining SA homeostasis, potentially through a mechanism linked to ROS homeostasis: when oxidative stress caused by ROS escalates, SA content rises; subsequently, endogenous SA mitigates oxidative stress by controlling ROS, after which SA levels are restored to a normal range. However, current evidence remains insufficient to fully elucidate the relationship between Si and SA. Moreover, Si-mediated SA signal transduction has not yet been reported. Future research directions of considerable promise include exploring the Si-mediated regulation of NPR genes and associated transcription factors, such as EIN, bZIP, WRKY, TGA, and ABF, in modulating salt stress tolerance. Although combined application of SA and Si has been documented to alleviate salt stress and promote plant growth (Mushtaq et al., 2022; Ceritoglu et al., 2023; Jamshidi Jam et al., 2023), the molecular mechanisms underlying the interplay between SA and other phytohormones and signaling molecules remain largely unclear.

Cytokinins (CKs) are phytohormones conventionally associated with plant development. In recent years, increasing evidence has highlighted their role in alleviating salt-induced senescence and sustaining crop yield under saline conditions (Melnikava et al., 2026; Thennakoon et al., 2026). In tomato, Si supplementation under salt stress elevated endogenous cytokinin levels and stimulated the expression of cytokinin-related genes. Notably, the ameliorative effect of Si on salt-triggered leaf senescence was abolished by treatment with a cytokinin biosynthesis inhibitor. To further dissect the relationship between Si and CKs, Si was supplied to *Arabidopsis* mutants (*ipt1,3,5,7*) that possess minimal cytokinin content; in these mutants, Si failed to rescue salt-induced senescence (Gou et al., 2022). Moreover, the capacity of Si to confer salt tolerance via cytokinin modulation has also been reported in cucumber (Zhu et al., 2020). Plants adapt to salt stress by modulating gibberellin (GA) levels at different growth stages (Yu et al., 2020). Several GA metabolism-related genes, such as *VvGA2ox7*, *GhGA2ox1*, and *AtGRF3*, have been reported to enhance salt tolerance by repressing growth (Shi et al., 2019; Gou et al., 2024; Le et al., 2026). An early study demonstrated that Si treatment increased GA content in soybean leaves under salt stress, and the GA biosynthetic pathway was identified as non-13-hydroxylation, leading to the formation of bioactive GA_4_ (Lee et al., 2010). Under salt stress, exogenous application of SiNPs upregulated the expression of GA biosynthesis genes (*GA20ox1* and *GA20ox2*) and downregulated GA inactivation genes (GA2ox1 and *GA2ox2*) in tomato seeds (Wang et al., 2024b). In cucumber seed germination assays, the addition of Si under salt stress did not alter the expression of genes encoding GA metabolism enzymes (*GA20ox*, *GA3ox*, and *GA2ox*) at 12 h after germination onset; however, at 36 h post-germination, Si suppressed the expression of the GA catabolic gene *GA2ox* (Gou et al., 2020). Furthermore, under 150 mM NaCl stress, 0.3 mM sodium silicate increased GA content in zucchini seedlings (Zhang et al., 2025b). Similar to other stress hormones, ethylene (ET) accumulates under salt stress, implying its essential role in salt responses. Further evidence demonstrated that the addition of 0.83 mM Si to sorghum seedlings subjected to 100 mM NaCl reduced the concentration of the ethylene precursor 1-aminocyclopropane-1-carboxylic acid (ACC) (Zhang et al., 2025b). However, a separate study reported that Si nanoparticles (SiNPs) increased the levels of ethylene, brassinosteroids, auxin, and cytokinins in *Elymus sibiricus* (L.) seedlings under saline conditions (Khan et al., 2024). Moreover, transcriptomic analysis revealed 435 differentially expressed genes (DEGs) between the combined NaCl+Si treatment and NaCl alone. The upregulated DEGs in the NaCl+Si treatment was enriched in functional categories associated with salt stress, ethylene and abscisic acid (ABA) biosynthesis, lignin catabolic processes, cell wall biosynthesis, and MAPK signaling pathways. Notably, exogenous Si application also upregulated the expression of transcription factors belonging to the AP2/ERF family (Wang et al., 2026d).

### 3.2 Coordination with other signaling molecules: building a rapid-response signaling microcircuit

#### 3.2.1 Calcium (Ca^2+^ Signals): master orchestrator of signal oscillation rhythms

Calcium ions (Ca^2+^) function as a universal primary second messenger in plant salt-stress sensing and signaling (Ma et al., 2019; Yang et al., 2019). It has been well documented that transient increases in cytosolic calcium levels serve as early signaling events in plant responses to abiotic and biotic stresses (Ji et al., 2024).The defining feature of Ca^2+^ signaling lies not in a simple elevation of cytosolic Ca^2+^ concentration but in the generation of oscillatory patterns characterized by specific frequencies, amplitudes, durations, and spatial waveforms (Ji et al., 2024; Lindberg and Premkumar, 2024). These dynamic Ca^2+^ signatures encode stress information and are subsequently decoded by downstream sensor proteins, thereby triggering distinct defense programs (Luan, 2026). A substantial body of evidence has demonstrated that Si application increases calcium content in plant tissues under salt stress (Avestan et al., 2021; Akhter et al., 2023; Aouz et al., 2023; Jam et al., 2023).

Investigations into cytosolic Na^+^ accumulation and the subcellular regulation of Na^+^ by Si have revealed that, under short-term salinity, Si supplementation reduced Na^+^ concentrations in both the apoplast and symplast of maize leaves, while concurrently elevating Ca^2+^ levels (Mahmood et al., 2024). In salt-stress studies, the rapid NaCl-induced elevation of cytosolic Ca^2+^ is decoded by a suite of Ca^2+^ sensors, including calmodulin (CaM), calmodulin-like proteins (CMLs), calcineurin B-like proteins (CBLs), CBL-interacting protein kinases (CIPKs), and calcium-dependent protein kinases (CDPKs) (Luan, 2026). Among these, CaM4 functions as a positive regulator of the Salt Overly Sensitive (SOS) pathway, acting in concert with SOS3 and SCaBP8 to modulate SOS2 kinase activity and thereby enhance salt tolerance in *Arabidopsis* (Zhang et al., 2025a). CBL5, which plays a role in seed germination, protects seeds and developing seedlings from salt stress through the CBL5–CIPK8/CIPK24–SOS1 axis (Xie et al., 2024). Under salinity, this enhanced decoding capacity promotes stronger activation of the SOS3–SOS2 (CBL4–CIPK24) kinase cascade, which in turn more effectively phosphorylates and activates the plasma membrane Na^+^/H^+^ antiporter SOS1, thereby accelerating Na^+^ efflux and restoring ionic homeostasis (Steinhorst et al., 2022). Furthermore, Si upregulates the expression of Na^+^ transporter genes, including SOS1 and NHX1, in apple under both short-term and long-term salt stress (Kılıç et al., 2023). However, the mechanisms by which Si mediates transcriptional regulation of salt adaptation through Ca^2+^ sensors and downstream signaling remain to be elucidated. In addition, salt-induced lipid peroxidation and membrane depolarization can impair Ca^2+^ channel activity. Given the fundamental role of Si in stabilizing membrane systems and the cell wall, whether Si can preserve the normal function of diverse Ca^2+^ channels localized in the plasma membrane, tonoplast, and endoplasmic reticulum under salt stress warrants further investigation.

#### 3.2.2 Nitric oxide: a synergistic amplifier in establishing antioxidant defense

Nitric oxide (NO), a key reactive nitrogen species and gaseous signaling molecule, plays a central role in plant stress perception, coordination of antioxidant defenses, and activation of systemic resistance (Ahmad et al., 2024a). In land plants, NO production involves multiple enzymatic and non-enzymatic routes. Nitrate reductase (NR)-dependent nitrite reduction represents one of the best-characterized enzymatic sources, whereas the molecular identity and contribution of any arginine-dependent NOS-like system remain unresolved (Jeandroz et al., 2016; Feng et al., 2019; Kishorekumar et al., 2020). NO alleviates oxidative damage by activating antioxidant enzymes and inhibiting peroxynitrite formation (Corpas and Barroso, 2015). In addition, NO contributes to salt stress mitigation by maintaining osmotic homeostasis (Huang et al., 2020).

Previous studies have reported that Si confers salt tolerance in soybean by modulating the crosstalk between antioxidants and NO levels (Chung et al., 2020). Under salt stress, genes associated with S-nitrosothiols and NO metabolism were upregulated, whereas their expression decreased following Si supplementation. Specifically, compared with NaCl treatment alone, the Si+NaCl treatment reduced S-nitrosothiol content by 24.2%. At 12 h post-stress initiation, all three S-nitrosothiol-related genes (*GmNOR1*, *GmNOR2*, and *GmNOR3*) were downregulated in Si-supplemented plants relative to those subjected to NaCl alone. Similarly, the activities of ascorbate peroxidase, peroxidase, and glutathione declined upon Si addition, and the expression levels of the antioxidant-related genes *GmCAT1*, *GmCAT2*, and *GmAPX1* began to decrease at 12 h after Si application. NR is a key enzyme responsible for NO production in plants (Chung et al., 2020). In okra (Abbas et al., 2017), rapeseed (Bybordi, 2012), and *Panicum turgidum* (Alabdallah et al., 2024) under salt stress, Si application increased NR activity. This Si-induced elevation of NR activity promoted NO accumulation, which likely laid the foundation for enhanced antioxidant enzyme activity.

### 3.3 Current limitations and unresolved questions in Si-mediated signal integration

Although the evidence reviewed above supports the conclusion that Si modifies phytohormone homeostasis, Ca^2+^-dependent responses, NO metabolism, and redox regulation under salinity, it does not yet establish Si as a direct molecular integrator of these signaling pathways. This distinction is important because most Si studies have measured hormone concentrations, total tissue Ca^2+^ contents, nitrate reductase activity, S-nitrosothiol pools, antioxidant activities, or stress-responsive transcript abundance several hours or days after salt exposure. These measurements capture the downstream physiological state of the plant but do not necessarily identify the primary signaling event through which Si acts. By contrast, studies of salt signaling in the absence of Si have resolved several upstream components with substantially greater spatial, temporal, and genetic precision. These include Na^+^ binding by plasma-membrane glycosyl inositol phosphorylceramide sphingolipids, MOCA1-dependent generation of cytosolic Ca^2+^ spikes and waves, a spatially restricted sodium-sensing niche in the root differentiation zone, and stress-intensity-dependent switching between CBL4- and CBL8-containing SOS modules. Therefore, the central limitation of the current Si literature is not a lack of signaling-associated phenotypes, but the absence of a clearly demonstrated causal connection between Si perception and the earliest salt-sensing machinery (Jiang et al., 2019; Steinhorst et al., 2022).

A major unresolved issue is the direction and temporal organization of Si-mediated hormonal regulation. Available results do not support a simple model in which Si consistently increases or suppresses a given phytohormone. In rice, short-term Si application increased ABA accumulation and the expression of several ABA-biosynthetic genes at 6–12 h, whereas these effects diminished or reversed by 24 h; JA was generally reduced, while SA showed a less consistent response (Kim et al., 2014). In tobacco, Si-induced ABA biosynthesis was associated with increased root hydraulic conductance, and inhibition of ABA synthesis weakened the hydraulic response to Si, providing partial functional evidence for an ABA-dependent mechanism (Liu et al., 2024). Recent work in cotton similarly linked an early ABA increase to stomatal closure and subsequent improvements in aquaporin abundance, root hydraulic conductance, and leaf water status, but the proposed pathway was inferred primarily from physiological measurements and RNA-seq rather than receptor or signaling-mutant analysis (Wang et al., 2026b). Conversely, after 75 d of salinity, Si maintained ABA, SA, and JA closer to control levels in grapevine, indicating that the long-term outcome may be hormonal normalization rather than sustained pathway activation (Urbano-Gálvez et al., 2025). Collectively, these findings support a model of time-dependent hormonal buffering or homeostatic tuning, but they do not yet define whether Si directly regulates hormone biosynthesis, perception, transport, conjugation, degradation, or feedback control.

The causal evidence is also uneven among different hormonal pathways. One of the strongest examples is the cytokinin study in tomato and Arabidopsis, in which inhibition of cytokinin synthesis abolished the Si effect and Si failed to delay salt-induced senescence in the cytokinin-deficient *ipt1,3,5,7* mutant (Gou et al., 2022). However, equivalent genetic evidence is largely unavailable for the major ABA, JA, SA, and ethylene signaling modules. Salt-stress research without Si has begun to use signaling mutants to resolve these relationships. For example, ABA-insensitive *SnRK2.2* and *SnRK2.3* plants exhibited prolonged Na^+^-specific transcriptional responses, increased cellular damage, impaired halotropic growth, and elevated shoot Na^+^ accumulation, demonstrating that ABA signaling gates rather than merely amplifies sodium-induced responses. Similarly, analysis of ethylene-deficient aco1a and jasmonate-deficient *lox3a* squash mutants showed that ABA–ET–JA interactions vary between germination and root growth and that the same hormone can exert different effects depending on developmental stage and tissue context (Alonso et al., 2024; Lamers et al., 2025). Comparable epistasis experiments have rarely been incorporated into Si–salinity studies. Future work should therefore test whether Si responses are retained or abolished in mutants or edited lines affecting *PYR/PYL*–*PP2C*–*SnRK2*, *COI1*–*JAZ*–*MYC2*, *NPR1*–*TGA*, and *ETR/EIN2/EIN3* signaling. Measurements should also distinguish bioactive JA-Ile from total JA, individual active cytokinin forms from the total cytokinin pool, ACC from ethylene production, and free ABA from conjugated and catabolic derivatives.

Evidence that Si directly reprograms Ca^2+^ signaling is particularly limited. An increase in total Ca concentration in a root or leaf does not demonstrate a change in the cytosolic Ca^2+^ signature that encodes salt information. Under salt stress alone, live-cell imaging has shown that Na^+^ induces Ca^2+^ signals with specific amplitudes, wave velocities, cellular origins, and dose dependencies. *MOCA1*-dependent GIPC sphingolipids participate in Na^+^ perception and Ca^2+^ influx, while increasing signal amplitudes recruit distinct Ca^2+^ decoders, including *CBL4* at moderate salinity and *CBL8* under more severe salinity. Additional genetic studies have identified tissue- and developmental-stage-specific modules such as *CBL5*–*CIPK8*/*CIPK24*–*SOS1* during seed germination (Jiang et al., 2019; Steinhorst et al., 2022; Xie et al., 2024). In comparison, most Si studies report changes in total Ca^2+^ content, expression of *SOS1* or *NHX* genes, membrane stability, or downstream ionic phenotypes. These findings are consistent with an influence of Si on Ca^2+^-dependent salt responses but do not establish that Si alters the initiation, amplitude, frequency, propagation, or decoding of Ca^2+^ signals. Direct evidence will require genetically encoded Ca^2+^ reporters, such as GCaMP- or cameleon-type sensors, combined with rapid imaging after NaCl exposure and genetic analysis using *moca1*, Ca^2+^-channel, *cbl*, *cipk*, and *sos* backgrounds. It will also be necessary to distinguish whether Si modifies channel gating directly, preserves Ca^2+^ signaling indirectly through membrane stabilization, or simply reduces the Na^+^ and ROS burden that would otherwise disrupt Ca^2+^ homeostasis.

The relationship between Si and NO is similarly unresolved and should not be described as unidirectional signal amplification. In salt-stressed soybean, Si reduced S-nitrosothiol accumulation and downregulated several NO-, reactive nitrogen species-, and antioxidant-related genes relative to salt treatment alone, which is compatible with relief of nitrosative and oxidative pressure rather than stimulation of a larger NO burst (Chung et al., 2020). By contrast, several physiological studies have reported increased nitrate reductase activity following Si treatment and have inferred increased NO generation. These apparently opposite results may represent different phases of the response: an early, spatially restricted NO signal may contribute to acclimation, whereas sustained NO or S-nitrosothiol accumulation may indicate continuing cellular stress. However, the relevant temporal sequence has not been resolved in Si-treated plants. Moreover, experiments involving the simultaneous application of Si and an NO donor can demonstrate compatibility or combined effectiveness but cannot, by themselves, prove that endogenous NO acts downstream of Si. Establishing such a relationship will require carefully controlled NO-donor and scavenger experiments, nitrate-reductase-deficient or GSNOR-altered genotypes, real-time and tissue-resolved NO detection, and identification of downstream S-nitrosylated proteins.

The biochemical origin of plant NO also requires more cautious treatment. A canonical animal-type nitric oxide synthase has not been identified in extensive surveys of land-plant transcriptomes; typical NOS-like sequences were absent from 1,087 examined land-plant transcriptomes but were detected in several algal lineages. Therefore, statements that Si regulates a defined plant “NOS pathway” are currently stronger than the available molecular evidence. In land plants, nitrate reductase-dependent nitrite reduction is one of the best-characterized enzymatic routes of NO production, but additional enzymatic and non-enzymatic sources may contribute depending on cellular compartment, oxygen availability, pH, and redox state. Consequently, future Si studies should attribute NO production to a specific pathway only when supported by genetic, biochemical, or isotope-tracing evidence rather than solely by sensitivity to mammalian NOS inhibitors (Jeandroz et al., 2016).

Another limitation is the lack of experiments that simultaneously resolve hormonal, Ca^2+^, NO, and ROS dynamics within a common temporal and spatial framework. Ca^2+^ and NO signals may arise within seconds to minutes, transcriptional responses occur over minutes to hours, and changes in hormone pools, ion accumulation, water status, and biomass may require hours to weeks. Measurements at one or two bulk-tissue sampling points cannot establish whether Ca^2+^ precedes ABA accumulation, whether NO acts upstream or downstream of ROS, or whether changes in JA and SA are primary signaling responses or secondary consequences of reduced injury. Transcriptomic associations are also insufficient to infer pathway activation because key signaling processes depend on protein phosphorylation, protein degradation, receptor–ligand interactions, channel gating, and post-translational modifications such as S-nitrosylation. A more rigorous strategy would combine live Ca^2+^ and NO imaging with targeted hormone profiling, phosphoproteomics, S-nitrosoproteomics, ion-flux measurements, and cell-type-resolved transcriptomics across seconds-, minutes-, hours-, and days-scale sampling points.

Experimental designs must also separate the different components of salinity. NaCl simultaneously imposes Na^+^ toxicity, Cl^-^ effects, osmotic stress, membrane depolarization, and secondary oxidative injury. Salt-only studies have used iso-osmotic sorbitol treatments and different ionic conditions to distinguish Na^+^-specific Ca^2+^ signaling from general osmotic responses, whereas many Si studies compare only control, NaCl, Si, and NaCl + Si treatments. Under such designs, it remains unclear whether Si modifies Na^+^ perception, osmotic signaling, membrane injury, or a downstream consequence shared by all these processes. Future experiments should include iso-osmotic non-ionic treatments, alternative sodium and chloride salts, and appropriate counter-ion controls. This is especially important because sodium, potassium, and calcium silicates introduce additional ions, while Si nanoparticles vary in size, surface charge, coating, dissolution rate, and cellular accessibility. These variables may influence hormone and ion signaling independently of the Si moiety itself. The distinction between soluble silicic acid, conventional silicates, and engineered Si nanoparticles should therefore be incorporated into mechanistic interpretation rather than treating all Si formulations as functionally equivalent. The importance of separating Na^+^-specific and osmotic signals is demonstrated by salt-perception studies in which MOCA1-dependent responses differed from sorbitol-induced osmotic signaling.

**Figure 3:**
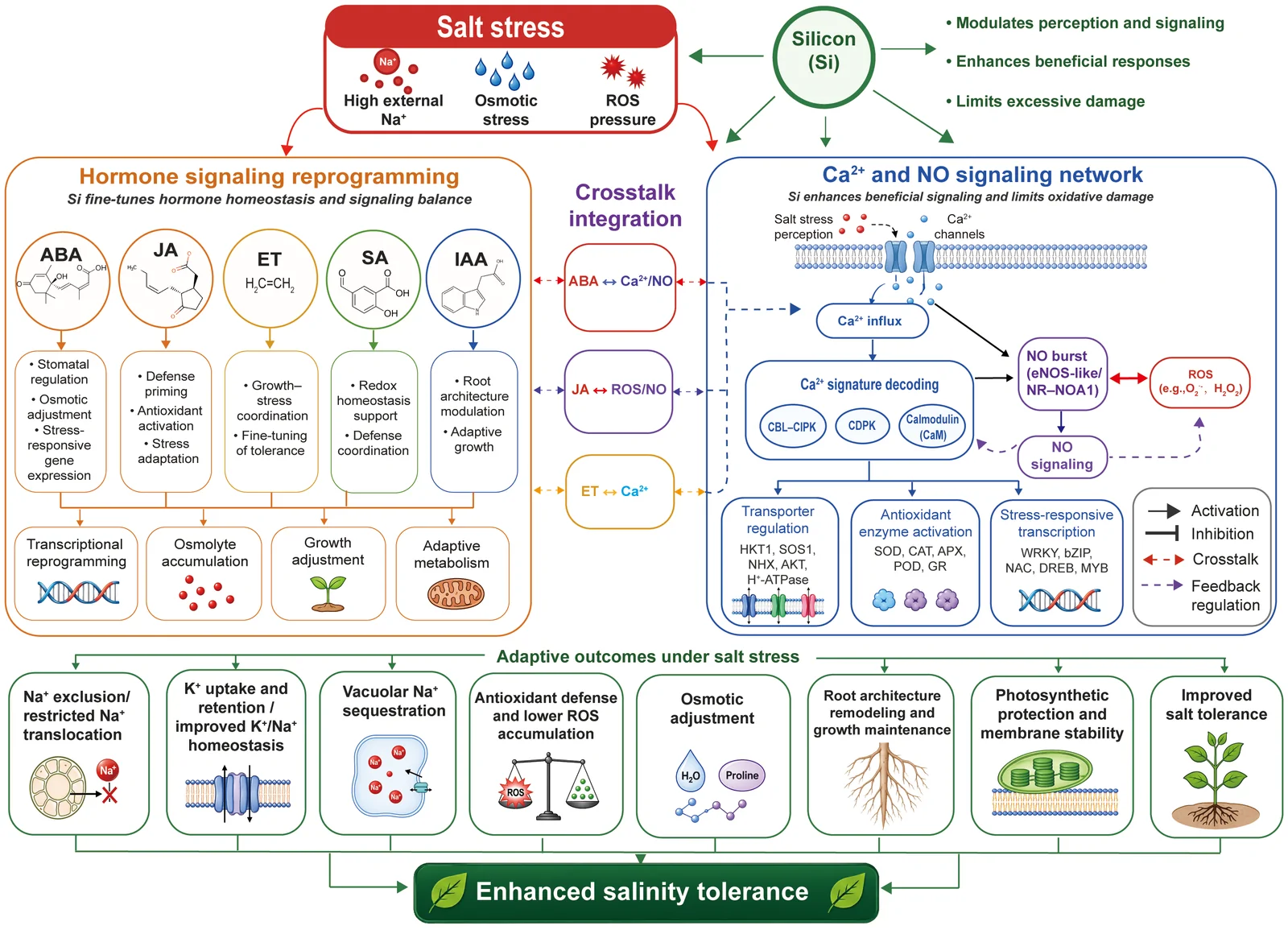
A proposed model of Si-associated hormonal, Ca^2+^, and NO signaling interactions during salt-stress adaptation. Salt stress perception activates interconnected hormonal and secondary messenger pathways, which are modulated by Si to optimize stress adaptation. Si treatment has been reported to alter phytohormone abundance and hormone-related gene expression, with consequences for stomatal regulation, osmotic adjustment, root development, and growth maintenance. Meanwhile, Si is proposed to influence Ca^2+^-dependent signaling by modifying membrane and ionic homeostasis and potentially affecting Ca^2+^ influx and downstream decoding through CBL–CIPK complexes, CDPKs, and calmodulin-dependent pathways. Ca^2+^ signaling interacts with NO and ROS signaling to modulate antioxidant defenses and stress-responsive gene expression. The integration of hormonal and Ca^2+^/NO pathways regulate ion transporters, antioxidant systems, transcriptional networks, and metabolic adjustment, ultimately enhancing cellular homeostasis and salt tolerance. Solid connections denote relationships supported by direct experimental evidence, whereas dashed connections represent proposed or indirect regulatory links that require further genetic and spatiotemporal validation.

Finally, the ecological and agronomic generality of the proposed signaling model remains uncertain. Most mechanistic experiments have been performed with seedlings in hydroponic or controlled pot systems, frequently using relatively high NaCl concentrations over short treatment periods. There is substantially less evidence from reproductive stages, gradual soil salinization, fluctuating field conditions, or multiseason yield experiments. Differences among Si accumulators and excluders, genotypes with contrasting Lsi transporter activity, plant developmental stages, tissues, and salt intensities may change both the magnitude and direction of hormone, Ca^2+^, and NO responses. Thus, **Figure 3** should be interpreted as a testable working framework rather than a completely established signaling pathway. The most defensible current conclusion is that Si alters the operating state, magnitude, and recovery kinetics of pre-existing salt-response networks. Whether Si directly initiates specific signals, modifies their encoding and decoding, or primarily buffers the cellular environment in which these signals operate remains unresolved. Resolving this distinction will require time-resolved genetics, live-cell reporters, pathway-specific perturbations, spatial multi-omics, and validation under field-relevant salinity regimes.

## 4 Core mechanism II: modulation of the rhizosphere microbiome

### 4.1 Modulation of rhizosphere metabolites by Si and its selective shaping of the microbial community

Under abiotic stress, metabolite–microbe interactions serve as a critical hub for sustaining crop productivity. Si mediates rhizosphere metabolic reprogramming and microbial modulation to synergistically enhance plant stress resilience (Yuan et al., 2022; Qin et al., 2026). In bacteria and archaea, Si can modify cell wall composition (Linklater et al., 2023), thereby disrupting membrane integrity and perturbing ionic homeostasis—particularly for essential divalent cations such as Mg^2+^ and Ca^2+^, which serve as critical cofactors for enzymes involved in DNA replication, protein synthesis, and energy metabolism (de Oliveira Bezerra et al., 2025). Such ionic perturbations may compromise enzymatic performance and consequently limit microbial growth efficiency. In fungi, Si may be deposited within the chitin–glucan matrix of the cell wall, altering permeability and interfering with nutrient transport as well as cellular signaling processes (Tripathi et al., 2021). Application of Si at 75 kg ha^-1^ significantly elevated the levels of nitrogenous and sugar-related metabolites in the rhizosphere, accompanied by changes in nitrogen-containing compounds such as urea. The enrichment of these metabolites provides readily accessible substrates and energy sources for rhizosphere microorganisms, thereby sustaining community activity and facilitating functional recovery (Qin et al., 2026). These results indicate that Si application modulates rhizosphere metabolic substrate supply and microbial assembly processes, thereby promoting the enrichment and activation of key functional microbial taxa, which consequently reinforces rhizosphere nutrient cycling and improves the sustainability and stability of nutrient supply. Principal coordinate analysis (PCoA) revealed that PCoA1 and PCoA2 explained 55.99% and 20.2% of the total variation, respectively, further demonstrating that Si fertilization exerts a marked influence on the diversity and composition of rhizosphere bacterial communities. In Si-amended soils, 475 microbial operational taxonomic units (OTUs) were significantly correlated with ethanamine, tagatose, urea, sorbose, and fumaric acid, among which OTU2080, OTU159, OTU194, OTU3246, and OTU1475 exhibited particularly strong correlations with these metabolites (Qin et al., 2026).

Compared with the control, low Si application rates (75 and 125 kg ha^-1^) significantly increased soil microbial biomass carbon by 53% and 44%, respectively, while also enhancing the activities of β-glucosidase (73%), alkaline phosphatase (115%), and arylsulfatase (68%) (Yuan et al., 2022). β-Glucosidase drives the depolymerization of polysaccharides into monosaccharides that sustain microbial metabolism (Sengupta et al., 2023); Si application appears to promote enzyme synthesis and catalytic efficiency, thereby accelerating organic matter decomposition and facilitating carbon turnover in the soil matrix. Notably, arylsulfatase activity was lowest at Si application rates of 0 and 225 kg ha^-^¹, suggesting inhibitory effects under both Si-deficient and Si-excess conditions (Cañizares et al., 2012; Zhang et al., 2024b). This trend may reflect Si-induced alterations in sulfur dynamics, given that arylsulfatase mediates the mineralization of organic sulfur compounds. Elevated Si levels may modify ion exchange and redox conditions, thereby reducing sulfate availability and enzyme activation (Yu et al., 2023). High-dose silicate may also impose osmotic stress and reshape microbial community structure, leading to the suppression of genes involved in sulfur cycling (Li et al., 2022; Yu et al., 2023). The aforementioned changes in metabolites and enzyme activities constitute important driving forces by which Si shapes rhizosphere microbial community structure. Si application exhibited a pronounced advantage in enhancing bacterial abundance within plants, and this effect displayed a distinct tissue-dependent distribution pattern (**Table 2**) (Qin et al., 2026).

**Table 2:** Effects of Si on bacterial abundance in different plant and soil compartments.

Compartment	Significantly increased	Significantly decreased
Leaf	*Cetobacterium, Bacteroides, Erysipelatoclostridium, Plesiomonas, Ruminococcaceae_NK4A214_group, Brevinema, Christensenellaceae_R_7_group, Edaphobacter, Aliidongia*	*Labedaea, Dokdonella, Crossiella, Haliangium*
Stem	*Lachnospiraceae_UCG_009*, *Blastococcus*, *Erysipelatoclostridium*, *Microbacterium*, *Allobaculum*, *Frondihabitans*, *Kineococcus*, *Ellin6067*, *Akkermansia*	*Gordonia, Yonghaparkia, Aquisphaera, Methylovirgula*
Root	*Acidiphilium*, *Fusobacterium*, *Allobaculum*	*Chujaibacter, Opitutus, Amycolatopsis, Haliangium, Bauldia, Saccharothrix*
Rhizosphere soil	*Mesorhizobium*, *Sinomonas*, *Enterobacter*, *Olivibacter*, *Glycomyces*, *Mucispirillum*, *Methylopila*	*Ochrobactrum, Amycolatopsis, Bacillus, Haematomicrobium, Alcaligenes, Aquicella, Corynebacterium_1, Acinetobacter, Brachybacterium, Lactobacillus, Leucobacter, Pseudomonas, Aeromonas, Brevibacterium, Akkermansia, Arthrobacter*
Bulk soil	*Blastococcus*, *Glycomyces*, *Kribbella*, *Massilia*, *Mucispirillum*	*Christensenellaceae_R_7_group, Saccharum hybrid cultivar*

The regulatory effects of Si on rhizosphere microbial communities have been further validated under various stress conditions (Ahmad et al., 2024b). Under low-temperature stress, Si-treated plants harbored significantly higher abundances of *Proteobacteria*, *Bacteroidota*, and *Ascomycota* compared with the control. The composition of the microbial community was positively correlated with extracellular enzyme activities in the soil and rhizosphere compartments. Under low-temperature stress, Si application increased extracellular enzyme activities, particularly those of phosphatase and glucosidase, most of which are produced by the microbial community (Ahmad et al., 2024b). These enzymes not only influence plant growth and development but also directly affect plant–microbe symbiosis. Under Cd–Pb combined stress, Si fertilization reduced the relative abundance of *Actinobacteria* while increasing that of *Bacteroidota*. The alpha diversity (Chao1 and Shannon indices) of bacterial communities in Si-treated soils was higher than that in the control, suggesting enhanced resistance of soil bacteria to combined Cd and Pb contamination (Wang et al., 2023). Si fertilization also modulated bacterial metabolic functions, with Si treatment leading to an increased abundance of bacteria associated with arginine and proline metabolism, glycolysis/gluconeogenesis, and the biosynthesis of valine, leucine, and isoleucine (Wang et al., 2023). The upregulation of these functions in soil could therefore increase and accelerate the decomposition of organic matter (Hou et al., 2018).A study investigating the rhizosphere microbial communities and metabolite profiles of an Al-sensitive species (*Eucalyptus tereticornis*) and an Al-tolerant species (*Eucalyptus urophylla*) under Si application revealed species-specific responses. Under Al+Si treatment, the levels of L-threonine, α-ketoisovaleric acid, 2-ketobutyric acid, isoleucine, ketoleucine, and L-valine metabolites were elevated in both species compared with Al treatment alone (Huang et al., 2026). Within the shared and significantly enriched valine, leucine, and isoleucine biosynthesis pathway, the accumulated metabolites may serve as an energy source for the rhizosphere system, enhance the activities of root antioxidant enzymes (e.g., SOD and POD), and facilitate Al chelation as well as metabolic homeostasis (Joshi et al., 2010). Additionally, the pentose phosphate pathway can generate NADPH, thereby providing robust reducing power to maintain redox homeostasis and support Al detoxification in plants (Stincone et al., 2015). In the Al-tolerant species, Si indirectly enhanced bacterial and metabolite diversity while recruiting specific microbial taxa, including *Candidatus Solibacter*, *Acidothermus*, and *Occallatibacter* (Huang et al., 2026). These Acidobacteria phylum microbes thrive in acidic soils and produce extracellular polysaccharides with hydroxyl (-OH) and carboxyl (-COOH) groups, which have been demonstrated to chelate Al and reduce its toxicity (Kalam et al., 2020; Larson et al., 2023).

Recent studies in salt-stressed soybean provide more direct evidence that Si-mediated metabolic reprogramming can guide the assembly of plant-associated microbial communities. Under 200 mM NaCl stress, pristine and functionalized SiO_2_ nanoparticles markedly altered root-exudate profiles, with increased levels of amino acids and signaling-related metabolites, including L-tyrosine, L-tryptophan, uridine, indole-3-acetamide, 3-indoleacetonitrile, proline, and L-arginine. Metabolite–microbe network analysis further linked these compounds to specific bacterial ASVs, with L-tryptophan showing predominantly positive associations with diverse bacterial lineages. These findings suggest that Si-induced changes in root exudation may function as selective chemical filters that regulate microbial community assembly under salinity (Chen et al., 2025). A complementary study showed that the spatial pattern of microbiome restructuring also depended on Si formulation: foliar SiO_2_ nanoparticles exerted stronger effects on leaf and root endophytic communities, whereas conventional silicate more strongly affected the rhizosphere bacterial community under 100 mM NaCl stress (Wang et al., 2024a). Thus, Si-mediated microbial selection is jointly determined by plant metabolic reprogramming, Si formulation, application route, and microbial compartment. More direct evidence for metabolite-mediated microbial recruitment under salinity was provided by Chen et al. (2026). In soybean exposed to 200 mM NaCl, the combined application of SiO_2_ nanoparticles and a functionally complementary synthetic community (SynC3) produced marked changes in root-exudate composition, with 5-aminovaleric acid (5-AVA) and succinic acid increasing by approximately 36.5- and 19.1-fold, respectively, compared with salt stress alone. Among the altered metabolites, 5-AVA showed a strong positive relationship with rhizosphere *Sphingomonas* abundance. Quantitative chemotaxis assays further demonstrated that *Sphingomonas paucimobilis* 436 was specifically attracted by 5-AVA. Exogenous 5-AVA application increased the absolute abundance of rhizosphere *Sphingomonas* by 33.9% and improved soybean growth under salt stress. These findings move beyond correlation and identify 5-AVA as a selective chemical cue linking Si-associated root metabolic reprogramming with the recruitment of a salt-beneficial bacterium (Chen et al., 2026).

Taken together, the selective influence of Si on rhizosphere and endophytic microbial communities is not unidimensional but arises from coordinated changes in root metabolism, exudate composition, rhizosphere physicochemical conditions, and microbial functional activity. This modulation leads to the consistent enrichment of particular functional assemblages (such as *Proteobacteria*, *Acidobacteria*, and beneficial genera) across stresses like chilling, heavy metal, and Al toxicity, alongside the suppression of certain potentially pathogenic taxa. The observed responses are dose- and species-dependent; nevertheless, they collectively underscore a central role of Si in ameliorating rhizosphere carbon and nitrogen substrate conditions, thereby bolstering microbial community functional resilience and promoting efficient nutrient turnover. Although most available studies remain descriptive or correlation-based, recent chemotaxis, isolate-validation, and synthetic-community experiments have begun to establish direct links between Si-associated root metabolites and the selective recruitment of beneficial microorganisms. Nevertheless, whether similar metabolite-guided recruitment mechanisms operate across crop species, Si formulations, and field environments remains unresolved.

### 4.2 From selection to activation: Si-mediated enrichment and functional stimulation of beneficial rhizosphere microbes

At the microbial level, Si fertilization significantly enhanced microbial diversity, community cohesion, and niche breadth, indicating an improved capacity of the microbial community to adapt to stress and maintain system stability, while selectively enriching specific functional taxa to activate key ecological processes (Qin et al., 2026). Si fertilization significantly increased the relative abundance of *Bacillus*, with OTU2080 (*Bacillus*) showing significant correlations with rhizosphere metabolites and nitrogen cycling-related indicators, suggesting its potential involvement in Si -driven rhizosphere functional reprogramming (Qin et al., 2026). In sugarcane rhizosphere soil, Si application significantly enriched *Mesorhizobium* and *Methylopila*; the former has been documented to promote legume growth and provide available nitrogen to plants in metal-contaminated environments (Drinkwater et al., 2017; Shahid et al., 2021), while the latter plays a crucial role in enhancing soil fertility and health, thereby boosting plant growth promotion and crop yield (Kumar et al., 2019). Under aluminum stress, Si further increased the abundance of diverse dominant bacterial and fungal genera, such as *Candidatus Koribacter*, *Occallatibacter*, *Candidatus Udaeobacter*, *Acidothermus*, and Ellin516, accompanied by a significant increase in plant growth-promoting rhizobacteria including *Azospirillum*, *Rhizomicrobium*, and *Sphingomonas* (Huang et al., 2026).

Under combined cadmium-lead stress, *Actinobacteria*, *Nitrospirae*, and *Sphingomonadales* were significantly enriched, while Si fertilization increased the abundance of *Betaproteobacteria* (Wang et al., 2023); these taxa may therefore serve as potential biomarkers for heavy metal tolerance in soils (Hicks et al., 2022). Under low-temperature stress, Si-treated roots and soil exhibited increased abundances of *Noviherbaspirillum* and *Bacillus* (Ahmad et al., 2024b), respectively. *Noviherbaspirillum* has previously been reported in cold-specific root microbiomes and other extreme environments, possessing plant stress tolerance-promoting properties (Zhang et al., 2021; Khan et al., 2022; Persyn et al., 2024), whereas *Bacillus* is recognized as a Si-solubilizing bacterium (SSB) that solubilizes Si from complex forms and renders it available for plant uptake (Vasanthi et al., 2018; Etesami and Jeong, 2022). In the rhizosphere of Lsi1-overexpressing rice plants, the abundances of bacteria involved in photosynthesis, energy metabolism, nitrogen fixation, and defense were significantly increased. In contrast, the populations of plant-associated pathogenic microorganisms, such as *Clostridium* and *Flavobacterium*, were markedly reduced, whereas beneficial taxa (e.g., *Lysobacter*), known for secreting antibiotics and bioactive substances, were enriched (Xie et al., 2022), thereby effectively suppressing pathogen invasion and safeguarding plant health (Bejarano et al., 2021; Jayasekera et al., 2025).

Salinity-specific studies further demonstrate that Si can enrich potentially beneficial bacterial guilds in a formulation- and compartment-dependent manner. In soybean exposed to 100 mM NaCl, foliar SiO_2_ nanoparticles preferentially enriched endophytic taxa, including Pseudomonas, Bacillus, Variovorax, and the *Allorhizobium*–*Neorhizobium*–*Pararhizobium*–*Rhizobium* group. SiO_2_ nanoparticle-enriched genera such as Variovorax and Bacillus were positively associated with root length, surface area, volume, and root-tip number, whereas changes in the leaf endophytic community were associated with photosynthetic performance and the shoot Na^+^/K^+^ ratio. Conventional silicate, by contrast, induced a stronger response in the rhizosphere community, indicating that different Si forms activate distinct microbial compartments (Wang et al., 2024a). Under 200 mM NaCl, carboxyl-functionalized SiO_2_ nanoparticles further enriched *Arthrobacter*, *Pseudomonas*, *Haliangium*, *Gemmatimonas*, and other potentially beneficial taxa, several of which were negatively correlated with the root Na^+^/K^+^ ratio (Chen et al., 2025).

Beyond taxonomic enrichment, SiO_2_-COOH treatment increased the predicted functional potential of rhizosphere bacteria related to Na^+^ extrusion, K^+^ uptake, arginine and proline metabolism, osmolyte accumulation, biofilm formation, and flagellar assembly. These changes were accompanied by increased soil NH_4_^+^-N, NO_3_^-^-N, available phosphorus, and organic matter, suggesting that Si-mediated microbial activation may contribute simultaneously to ionic regulation, osmotic adjustment, and nutrient acquisition under salinity (Chen et al., 2025). However, because these functions were inferred primarily from 16S rRNA-based PICRUSt2 prediction, they should be interpreted as putative functional potentials rather than directly measured gene expression or microbial activity.

Recent salinity-specific evidence further demonstrates that Si-associated microbial selection can be translated into experimentally validated microbial functions. Under 200 mM NaCl, SynC3 alone enriched *Lysobacter*, *Streptomyces*, and *Nocardioides*, whereas SiO_2_ nanoparticles enriched *Sphingomonas*, MND1, and RB41. Their combined application preferentially enriched *Sphingomonas*, *Streptomyces*, and Ellin6055, with *Sphingomonas* accounting for 16.2% of the rhizosphere bacterial community. Shotgun metagenomic analysis showed that the combined treatment increased the abundance of microbial genes associated with Na^+^ extrusion, K^+^ uptake, nicotinate and nicotinamide metabolism, and osmotic adaptation. In addition, inoculation with *S. paucimobilis* 436 promoted soybean growth in a sterile substrate under salt stress, providing direct functional support for the contribution of this Si-associated taxon to plant salt tolerance (Chen et al., 2026). Evidence from drought-stressed wheat indicates that Si-mediated microbial activation is not restricted to salinity. Seed nanopriming with 500 mg L^-1^ Si quantum dots substantially reshaped the wheat rhizosphere bacterial community and partially restored the abundance of Proteobacteria that had declined under drought. Si quantum dots selectively enriched *Luteimonas*, *Arenimonas*, and *Lysobacter* and restored beneficial genera including *Sphingomonas*, *Variovorax*, *Sphingosinicella*, and *Mesorhizobium*. Metagenomic analysis further revealed enrichment of genes involved in lipopolysaccharide biosynthesis, biofilm formation, and bacterial secretion systems. These microbial functions may enhance rhizosphere colonization, extracellular polymeric substance production, microbial communication, and community stability under water deficit. The microbial changes occurred together with increased wheat biomass, relative water content, proline and soluble sugar accumulation, and reduced lipid peroxidation, supporting a coordinated physiological–microbial contribution to drought tolerance (Meng et al., 2026). The two studies also illustrate different levels of mechanistic evidence. Chen et al. (2026) combined metagenomics with chemotaxis assays, isolate inoculation, and SynC validation, whereas Meng et al. (2026) linked metagenomic functional enrichment with plant physiological responses but did not directly test whether the altered microbiome was necessary for the SiQD-induced drought-tolerant phenotype.

These enriched beneficial microorganisms directly stimulate nutrient transformation and plant growth-promoting functions through multiple mechanisms: *Azospirillum* and *Mesorhizobium* contribute to biological nitrogen fixation, supplying nitrogen nutrition to plants; *Bacillus* and *Sphingomonas* promote the dissolution of Si and phosphorus as well as the chelation and passivation of metal ions through organic acid secretion; and *Lysobacter* inhibits pathogen colonization and spread by producing antibiotics and bioactive compounds. Meanwhile, Si fertilization upregulated microbial functions associated with arginine and proline metabolism, glycolysis/gluconeogenesis, and valine, leucine, and isoleucine biosynthesis, accelerating organic matter decomposition and nutrient turnover in soils (Hou et al., 2018). Throughout this process, Si optimizes rhizosphere carbon and nitrogen substrate availability and modulates metabolic pathways, creating favorable micro-environmental conditions for the enrichment and functional expression of beneficial microorganisms, ultimately bolstering microbial community functional resilience and nutrient cycling efficiency, and providing sustained ecological functional support for plant growth under stress conditions. To synthesize the emerging evidence discussed above, **Table 3** summarizes representative microbial taxa and functional guilds associated with Si-mediated alleviation of abiotic stress. Salinity-specific studies are presented first, followed by supporting evidence from cold, drought, metal, and other stress systems.

**Table 3:** Si-mediated changes in microbial communities and their potential roles in alleviating plant stress.

Stress type	Plant or experimental system	Si treatment	Si-mediated microbial changes	Major microbial functions	Stress alleviation and plant responses	Nature of evidence	Representative reference
Salinity	Soybean (*Glycine max* L*.)*	Foliar application of SiO_2_ nanoparticles or potassium silicate under 100 mM NaCl; final dose of 12 mg SiO_2_ plant^-1^ over 15 days	SiO_2_ nanoparticles exerted stronger effects on leaf and root endophytic communities, whereas potassium silicate more strongly affected the rhizosphere. SiO_2_ nanoparticles enriched potentially beneficial endophytic genera, including *Pseudomonas*, *Bacillus*, and *Variovorax*	Putative plant growth promotion and regulation of root development, photosynthetic performance, and ion homeostasis; functions were inferred mainly from taxonomic identity and plant–microbiome correlations	Improved root architecture and photosynthetic performance and reduced shoot and root Na^+^/K^+^ ratios	16S rRNA profiling and correlation analysis; no microbiome depletion, isolate re-inoculation, or synthetic-community validation	(Wang et al., 2024a)
Salinity	Soybean (*Glycine max*)	Pristine SiO_2_, amino-functionalized SiO_2_-NH_3_, and carboxyl-functionalized SiO_2_-COOH nanoparticles under 200 mM NaCl	Enrichment of *Variovorax* and *Pseudomonas* in the root endosphere and *Arthrobacter*, *Haliangium*, *Gemmatimonas*, RB41, and related taxa in the rhizosphere	Predicted Na^+^ extrusion, K^+^ uptake, osmolyte metabolism, biofilm formation, nutrient cycling, and phytohormone-related functions; accompanied by reprogramming of root exudates	Reduced Na^+^/K^+^ ratio, enhanced root growth and photosynthesis, and increased soil N and P availability	Root-exudate metabolomics, 16S rRNA sequencing, correlation networks, and PICRUSt2 functional prediction; microbial functions were not directly measured	(Chen et al., 2025)
Salinity	Soybean (*Glycine max*)	Soil application of 1 g kg^-^¹ SiO_2_ nanoparticles alone or combined with the functionally complementary SynC3 under 200 mM NaCl	The combined treatment enriched *Sphingomonas* and other SynC-associated taxa. It markedly increased root-derived 5-aminovaleric acid, which acted as a chemotactic signal for *Sphingomonas paucimobilis* 436	Shotgun metagenomics identified increased microbial genetic capacity for Na^+^ extrusion and K^+^ uptake. *Sphingomonas* contributed substantially to the *nhaA* and *kup* genes. Chemotaxis, rhizosphere recruitment, and the plant-growth-promoting function of *S. paucimobilis* 436 were experimentally validated	Increased biomass, proline accumulation, and antioxidant enzyme activities and reduced Na^+^/K^+^ ratios and ROS accumulation. In sterile substrate, *S. paucimobilis* 436 increased shoot and root fresh weights by 47.6% and 61.2%, respectively	16S profiling, shotgun metagenomics, root-exudate metabolomics, SynC construction, chemotaxis assays, absolute microbial quantification, sterile-substrate inoculation, and metabolite validation; strongest causal evidence among the included studies	(Chen et al., 2026)
Cold stress	Soybean (*Glycine max* cv. Fiskeby V)	Application of 1.0 mM silicic acid, approximately 1 g Si kg^-1^ soil, followed by exposure to 8–10 °C for two weeks	Si altered bacterial and fungal community composition across leaves, roots, and rhizosphere soil. Si-treated plants under cold stress showed increased abundances of *Noviherbaspirillum* in roots, *Bacillus* in soil, and *Sphingomonas* in leaf and root compartments; Proteobacteria, Bacteroidota, and Ascomycota were prominent Si-responsive phyla	Increased extracellular phosphatase and glucosidase activities, indicating enhanced microbial nutrient-mobilization potential; stress-adaptive functions of individual taxa were mainly inferred from previous studies	Increased biomass and shoot and root growth, reduced cold-induced oxidative stress, and altered expression of ABA- and dehydration-related genes	Factorial pot experiment with 16S and ITS profiling, extracellular enzyme assays, plant physiology, and gene-expression analysis; no microbial re-inoculation or depletion experiment	(Ahmad et al., 2024b)
Drought	Upland rice (*Oryza sativa* cv. Hanyou 73)	Field application of sodium silicate at 25–100 kg ha^-1^ under drought; the optimal treatment was 75 kg ha^-1^	Si increased bacterial richness and community niche breadth. The relative abundances of *Bacillus*, *Rhodanobacter*, and *Sphingomonas* increased; OTU2080 affiliated with *Bacillus* and OTU194 affiliated with *Rhodanobacter* increased by 100.0% and 80.31%, respectively. The enriched strain BT021 was identified as *Bacillus tropicus*	16S/KEGG annotation indicated increased potential for N fixation and nitrification and reduced denitrification and N_2_O-producing pathways. Soil urease and nitrate-reductase activities were directly increased. Si plus N- and sugar-related metabolites stimulated BT021 proliferation	Increased nitrogen-use efficiency by 26.21%, leaf SOD activity by 40.31%, and grain yield by 22.98% and 20.90% over two years; improved soil N supply, photosynthesis, antioxidant capacity, and yield stability under drought	Two-year field trial, metabolomics, 16S profiling, KEGG annotation, soil enzyme assays, sterile-substrate comparison, metabolite validation, and isolate re-inoculation; microbial dependence was supported experimentally	(Qin et al., 2026)
Drought	Wheat (*Triticum aestivum*)	Seed nanopriming with 500 mg L^-1^ silicon quantum dots under drought at 40% field capacity	silicon quantum dots (SiQDs) enriched or restored *Sphingomonas*, *Lysobacter*, *Variovorax*, *Luteimonas*, *Arenimonas*, *Sphingosinicella*, and *Mesorhizobium* in the rhizosphere	Shotgun metagenomics showed enrichment of genes and pathways involved in lipopolysaccharide biosynthesis, biofilm formation, extracellular polymeric-substance production, bacterial secretion, adhesion, and colonization. Major gene contributors included *Lysobacter*, *Arenimonas*, *Sphingomonas*, and *Variovorax*	Increased germination, shoot biomass, relative water content, proline, and soluble sugars and reduced root MDA; 500 mg L^-^¹ SiQDs increased shoot biomass by 157.1% and relative water content by 26.7%	16S rRNA profiling, shotgun metagenomics, and plant–microbiome correlation analysis; no microbiome depletion or isolate re-inoculation	(Meng et al., 2026)
Field drought	Winter wheat (*Triticum aestivum* L. cv. *Tybalt*)	Field incorporation of amorphous silica (Aerosil 300) at 0.5% or 1% into the upper 25 cm of soil before sowing during a dry growing season	ASi significantly altered bacterial and fungal community composition without changing overall microbial richness, diversity, or total bacterial and fungal abundance. The 1% ASi treatment enriched bacterial taxa including Acidobacteriota, WPS-2, Thermoleophilia, Frankiales, Planctomycetes, *Acidothermus*, *Arthrobacter*, *Conexibacter*, *Flexivirga*, and *Acidipila*. The 0.5% treatment enriched Xanthomonadales, Sphingomonadales, *Arenimonas*, and *Sphingomonas*. ASi also enriched potentially beneficial fungi, including *Dichotomopilus*, *Auxarthron*, and *Cladosporium*, in a dose- and growth-stage-dependent manner	*Arthrobacter* and *Sphingomonas* were associated with putative phytohormone production, nutrient acquisition, and abiotic-stress adaptation, while *Sphingomonas* may improve Fe acquisition through siderophore production. Enriched fungal taxa such as *Dichotomopilus*, *Auxarthron*, and *Cladosporium* were assigned putative plant-growth-promoting and biocontrol functions based on previously reported traits	The 1% ASi treatment significantly increased soil moisture throughout the investigated growth stages, whereas 0.5% ASi increased moisture mainly at tillering. ASi also temporarily increased available soil P and K. These changes may improve soil water and nutrient availability during drought, but the microbial contribution to wheat drought tolerance was not independently quantified	Field experiment combining soil physicochemical measurements, bacterial 16S and fungal ITS metabarcoding, qPCR, LEfSe, and co-occurrence-network analysis. Microbial functions were inferred from taxonomic identity and previous literature; no functional-gene analysis, microbial depletion, isolate re-inoculation, or direct microbiome-causality experiment was conducted	(Lewin et al., 2024)
Multiple heavy-metal stress	Pakchoi (*Brassica chinensis*) grown in soil contaminated with Pb, Cd, Cr, As, Hg, and Cu	Application of a slag-derived granular Si fertilizer at 0.2–3.2% (w/w); the strongest bacterial richness response occurred at 0.8%	The 0.8% treatment increased Chao and ACE richness, but Shannon diversity did not change significantly. *Nitrospirae*, *Flavobacteriia*, *Anaerolineae*, *Gemmatimonadales*, *Bacillales*, and *Betaproteobacteria* were more abundant in Si-treated soils. In contrast, putatively metal-resistant *Actinobacteria*, *Cyanobacteria*, and Cd-sensitive *Sphingomonadales* were more abundant in the untreated control	Potential involvement in soil C and N metabolism was inferred from the known ecological attributes of *Nitrospirae*, *Flavobacteriia*, *Anaerolineae*, and *Bacillales*. No microbial functional genes, microbial enzyme activities, or isolate functions were directly measured. Community changes were closely associated with Si-induced changes in soil pH, electrical conductivity, and available K	Increased pakchoi biomass and nutrient uptake. Depending on Si dose, tissue concentrations of Pb, Cr, Cu, Hg, and As were reduced, whereas Cd concentration was not significantly affected. Total metal uptake initially increased with biomass and then decreased at higher Si doses; therefore, the result should not be described simply as reduced heavy-metal accumulation	Pot experiment with 16S sequencing, LEfSe, NMDS, Mantel tests, and environmental correlations. Functions were taxonomically inferred; the slag fertilizer contained Ca, Mg, Fe, Mn, and other components that may have contributed to the response	(Wang et al., 2020)
Arsenic stress	Japonica and Indica rice grown in As-enriched paddy soil	Application of granular calcium silicate fertilizer at 2 Mg ha^-1^	Bacterial α-diversity was not significantly altered. *Gemmatimonadetes* increased in both cultivars. The genera *Bacillus*, *Ramlibacter*, *Geobacter*, and *Azospirillum* increased in both Japonica and Indica rice, whereas several other taxa responded in a cultivar-dependent manner	GeoChip analysis showed increased microbial genetic potential for oxidative-stress tolerance (*ahpC*, *ahpE*, *katA*, *katE*), osmotic-stress tolerance (*opuE*, *proV*), oxygen-limitation responses (*arcA*, *narH*), N- and P-limitation responses (*glnA*, *glnR*, *phoA*, *phoB*, *pstA–C*), and heat-, cold-, and radiation-stress responses. Activities of soil enzymes involved in labile C degradation and N and P cycling were also directly increased	Increased photosynthetic rate, transpiration efficiency, stomatal conductance, nutrient uptake, and crop yield. Grain As concentrations decreased by 29.8% and 26.1%, and straw as concentrations decreased by 44.4% and 51.4% in Japonica and Indica rice, respectively; grain yield increased by 27.8% and 22.7%	Greenhouse pot experiment using 16S profiling, GeoChip functional-gene microarray, soil enzyme assays, pore-water chemistry, and plant traits. GeoChip reflects functional-gene abundance or genetic potential, not gene expression; microbial causality was not tested	(Das et al., 2021)

Microbial functions inferred from taxonomic identity, correlation networks, or 16S rRNA-based functional prediction are regarded as putative unless validated by direct functional assays, isolate re-inoculation, microbiome depletion, microbiome transplantation, or synthetic-community experiments.

### 4.3 Proposed mechanisms of coordinated Si–plant–microbe interactions

Si, plants, and rhizosphere microorganisms may form coordinated interactions through changes in root exudation, microbial community assembly, nutrient cycling, and stress-related physiological responses. The coordinated outcomes of this three-way interaction cannot be fully explained by isolated plant- or microbiome-centered mechanisms and may provide an important ecological basis for maintaining plant productivity under stress.

At the signaling level, Si intervention initiates bidirectional reprogramming of plant–microbe communication. On one hand, Si treatment has been associated with changes in the quantity and composition of root exudates, which may influence microbial recruitment and community assembly (Huang et al., 2026; Qin et al., 2026), which not only provide chemical cues for the directed recruitment of rhizosphere microorganisms but also directly shape the trajectory of community assembly. On the other hand, the recruited beneficial microbes, such as *Pseudomonas* and *Azospirillum*, reciprocally modulate plant gene expression by secreting signaling molecules including salicylic acid and jasmonic acid, thereby activating transcription factor families such as MYB and bHLH, and promoting the synthesis of organic acids and defense-related secondary metabolites (Vacheron et al., 2013; Chauhan et al., 2021). This bidirectional signal reinforcement, from plant to microbe and from microbe to plant, constitutes a positive-feedback amplification loop, potentially reinforcing stress adaptation through coordinated plant-to-microbe and microbe-to-plant feedback (Huang et al., 2026; Qin et al., 2026).

Recent evidence provides an experimentally supported example of the Si–plant–microbe tripartite pathway under salinity. In salt-stressed soybean, SiO_2_ nanoparticles and the functionally complementary SynC3 jointly reprogrammed root-exudate composition and rhizosphere microbiome assembly. The combined treatment promoted the accumulation of 5-AVA, which acted as a chemotactic signal for the recruitment of *Sphingomonas*. The recruited *Sphingomonas* population and other SynC-associated microorganisms were linked to enhanced microbial Na^+^ extrusion, K^+^ uptake, nicotinate and nicotinamide metabolism, osmotic adjustment, and redox protection. At the plant level, the combined treatment increased root biomass, proline accumulation, and antioxidant enzyme activities while reducing Na^+^/K^+^ ratios and ROS accumulation more strongly than either SiO_2_ nanoparticles or SynC3 alone. This establishes a mechanistic sequence in which a Si-containing intervention modifies plant metabolic output, the altered metabolite environment recruits functional microorganisms, and microbial activities subsequently reinforce plant ionic, osmotic, and redox homeostasis (Chen et al., 2026).

A complementary study in drought-stressed wheat suggests that this ecosystem-level mode of action may extend beyond salinity. Seed-delivered Si quantum dots simultaneously improved plant water status, osmotic adjustment, antioxidant protection, and root development while restructuring the rhizosphere microbiome and activating microbial pathways associated with biofilm formation, bacterial secretion, and colonization. The recurrence of *Sphingomonas*, *Lysobacter*, and *Variovorax* across taxonomic enrichment and functional-gene contribution further suggests that certain microbial guilds may represent conserved components of Si-mediated stress adaptation across different environmental constraints (Meng et al., 2026). Nevertheless, direct microbiome-depletion or reciprocal-transplantation experiments are still required to establish whether these microbial changes are indispensable for the SiQD-induced drought-tolerant phenotype.

At the functional level, Si and beneficial microorganisms establish a clear division of labor and deep complementarity in nutrient acquisition, stress defense, and metabolic regulation. In some experimental systems, Si treatment has been associated with increased relative abundance of taxa such as *Azospirillum* and *Mesorhizobium*. These genera include strains with known nitrogen-fixing capacity, although their actual contribution to plant N acquisition following Si application has rarely been quantified directly (Drinkwater et al., 2017; Fukami et al., 2018; Shahid et al., 2021), while *Methylopila* synergistically enhances crop yield by improving soil fertility and health (Kumar et al., 2019). Simultaneously, Si upregulates the microbial functional potential associated with nitrogen cycling, especially nitrogen fixation and nitrification, and metabolically supports this process through the accumulation of nitrogenous sugars and amino acid metabolites (Qin et al., 2026). In metal detoxification and element mobilization, *Bacillus* and *Sphingomonas*, acting as SSB, secrete organic acids that convert insoluble Si into plant-available forms while simultaneously chelating and passivating heavy metal ions (Vasanthi et al., 2018; Etesami and Jeong, 2022; Wang et al., 2022). Si itself further reinforces this chelation by inducing the accumulation of organic acids and phenolic compounds in root exudates (Kidd et al., 2001; Huang et al., 2026). In regulating redox homeostasis, Si treatment has frequently been associated with increased activities of antioxidant enzymes, including SOD, POD, and CAT, although the response depends on stress intensity, tissue, and sampling time (Yuan et al., 2022), whereas enriched microbes such as Bacillus and *Noviherbaspirillum* synergistically reduce oxidative damage by secreting antioxidative metabolites or by inducing the synthesis of endogenous antioxidants in plants (Zhang et al., 2021). Moreover, Si application upregulates microbial metabolic functions, as evidenced by enhanced arginine/proline metabolism, glycolysis/gluconeogenesis, and the valine/leucine/isoleucine biosynthesis pathway (Wang et al., 2023). This upregulation further accelerates soil organic matter decomposition and nutrient turnover, thereby providing sustained energy and substrate support for the tripartite interaction (Hou et al., 2018). In terms of pathogen suppression, Si application or genetic modification (e.g., *Lsi1* overexpression) enriches *Lysobacter*, which secretes antibiotics and bioactive substances to inhibit the colonization and spread of pathogens such as *Clostridium* and *Flavobacterium* (Xie et al., 2022), thus offering an additional protective layer against biotic stress. Collectively, this multi-level functional complementarity forms a continuous and robust defense-nutrition chain from the rhizosphere to the whole plant.

At the ecological level, the three components form a self-reinforcing and sustainable system through niche optimization and nutrient cycling. Si regulates the availability of carbon and nitrogen substrates and extracellular enzyme activities (e.g., β-glucosidase, alkaline phosphatase, and arylsulfatase) in the rhizosphere (Yuan et al., 2022), thereby creating differentiated niches and competitive advantages for specific functional microbes. The enriched functional microbes, through activities such as organic matter decomposition, nitrogen fixation, and phosphate solubilization, further enrich the rhizosphere nutrient pool, which in turn supports plant growth and root metabolism, propelling the system into a virtuous cycle of metabolite enrichment, microbial activation, enhanced nutrient turnover, and plant growth promotion. Notably, Si-mediated tripartite interactions play critical roles in both the “selection” and “activation” stages of microbial community modulation. On one hand, Si selectively recruits beneficial functional taxa from the soil microbial reservoir via metabolic signals (i.e., the “selection” phase) (Qin et al., 2026). On the other hand, Si promotes the functional expression and metabolic activity of already enriched microbes (i.e., the “activation” phase) by creating favorable rhizosphere microenvironments, including optimized carbon/nitrogen substrates and modulated enzyme activities (Hou et al., 2018; Yuan et al., 2022). This dual-phase driving model, encompassing both selection and activation, endows the system with greater functional resilience to environmental fluctuations. Co-occurrence network analysis further reveals that Si addition enhances the connectivity and modularity within bacterial communities, thereby improving community stability and functional redundancy (Zheng et al., 2018), which provides a structural basis for rapid functional recovery after disturbance.

Notably, the effectiveness of the tripartite synergistic mechanism is subject to a clear dosage window and species specificity. At the dosage level, moderate Si supply (75–125 kg ha^-1^) maximizes the beneficial effects of the three-way interaction, with microbial biomass carbon, extracellular enzyme activities, and nitrogen-cycling potential all peaking within this range. In contrast, insufficient (0 kg ha^-1^) or excessive (≥225 kg ha^-1^) application may disrupt this balance and even impose metabolic stress on the microbial community (Yuan et al., 2022; Wang et al., 2023). At the species level, aluminum-sensitive and aluminum-tolerant species exhibit markedly different dependencies on the synergistic strategy of tripartite interactions. The former relies on Si to block aluminum entry at the rhizosphere through an exclusion-based mechanism, whereas the latter depends on coordinated internal homeostasis regulation (Huang et al., 2026). Similarly, a comparison between rice overexpressing *Lsi1* and its wild-type counterpart reveals that genotypic differences determine the plant’s capacity to recruit and sustain beneficial microorganisms (Xie et al., 2022). This divergence in strategies implies that the Si-plant-microbe synergy follows an optimal adaptation pathway dictated by plant genotype, offering important insights for precision Si fertilization and genotype-microbiome compatibility management.

**Figure 4:**
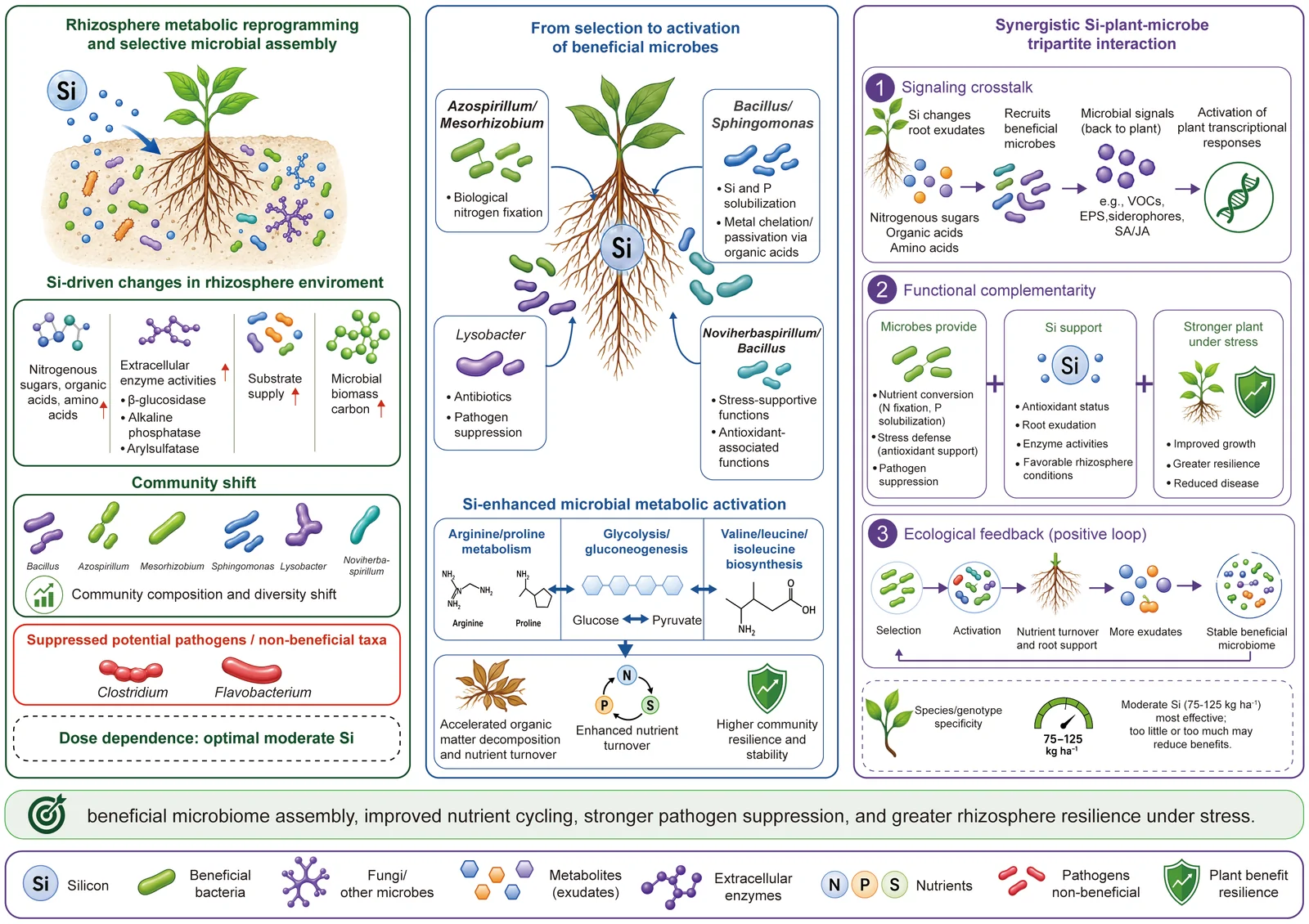
Si-mediated plant–microbiome interactions enhance plant resilience under environmental stress. Si application reshapes rhizosphere microbial communities by enriching beneficial microorganisms, improving nutrient cycling, regulating stress-related metabolites, and strengthening plant physiological adaptation under adverse environments.

In conclusion, the Si–plant–microbe tripartite interaction operates through signal crosstalk, functional complementarity, and ecological cycling to form a composite defense system driven by positive feedback, functional redundancy, and environmental adaptability. Based on the available evidence, we propose a two-stage conceptual model in which Si-associated changes in root metabolites and rhizosphere conditions may first influence microbial selection and subsequently affect the functional potential of the assembled community. This framework remains to be tested through microbiome depletion, transplantation, isolate re-inoculation, and synthetic-community experiments across different crops and soil environments. Future priorities include: (1) elucidating the molecular integration of Si signals with plant immune and microbial pathways; (2) quantifying the net contributions of tripartite interactions to C/N cycling and energy flux under varying environments; and (3) clarifying how genotypic differences shape interaction strategies to guide genotype-specific Si-inoculant formulations. The signaling crosstalk, functional complementarity, and ecological feedback loops underlying this ecosystem-level framework are synthesized in **Figure 4**.

To integrate the species-specific evidence discussed above, **Table 4** summarizes the major effects of Si on plant salt tolerance across five interconnected dimensions, including ion regulation, hormonal signaling, antioxidant responses, gene regulation, and plant–microbe interactions, together with their underlying mechanisms and reported physiological or molecular outcomes.

**Table 4:** Species-specific evidence for Si-mediated enhancement of plant salt tolerance through ion regulation, hormonal signaling, antioxidant responses, gene regulation, and plant–microbe interactions.

Resistance mechanism	Plant species	Silicon treatment and experimental setup	Response	Reference(s)
Regulate ion homeostasis	Cucumber (*Cucumis sativus* L., cv. *9930*)	Hydroponic experiment; control (CK), 100 mM NaCl (Na), 100 mM NaCl + 1.5 mM Na_3_SiO_3_ (Na+Si1), 100 mM NaCl + 3 mM Na_3_SiO_3_ (Na+Si2), 100 mM NaCl + 1.5 mM SiNPs (Na+SiNPs1), and 100 mM NaCl + 3 mM SiNPs (Na+SiNPs2).	Shoot: Na^+^ (-) 19.5-35.1% K^+^ (+) 20.4-37.1% Root: Na^+^ (-) 21.5-31.3% K^+^ (+) 21.2-37.6%	(Dong et al., 2026)
	Cluster bean (*Cyamopsis tetragonoloba* L.)	Pot experiment; salinity stress (0, 6, and 12 dS m^−1^) silicon dioxide nanopowder (50 mg L^−1^)	Na^+^ (-) 53.3-60.5% K^+^ (+) 87.7-100.1% K^+^/Na^+^ (+) 4- and 5.1-fold	(Rahimi et al., 2026)
	maize (*Zea mays* L., Monsento-6317 and SF9515)	Pot experiment; 100 ppm salinity; 500 ppm Na_2_O_3_Si·5 H_2_O	N (+) 7.90-8.37% P (+) 16.67%-21.9% K (+) 76.33%-111.47% Zn (+) 22.85-23.79% Cu (+) 46.51-58.65% Fe (+) 18.62-20.26% Mn (+) 6.96-9.07% B (+) 27.12-30.65% Na (-) 56.06-57.36%	(Ahmad et al., 2026)
	olive (*Olea europaea* L.)	Pot experiment; 100 mM NaCl; 10 mg L^-1^ Si(OH)_4_	New leaves: Na (-) 49-58%	(Fidalgo-Illesca et al., 2025)
	Rice (*Oryza sativa* L.)	Hydroponic experiment; 2 mmol L^-1^ nano-silica, 60 mmol L^-1^ NaCl	Leaves: Na (-) 13.29% Cl^-^ (-) 23.24% Si^4+^ (+) 73.84% K (+) 6.89% Ca^2+^ (+) 7.91% Na/K (+) 18.89% Roots: Na (-) 7.12% Cl^-^ (-) 4.04% Si^4+^ (+) 59.28% K (+) 9.15% Ca^2+^ (+) 82.20% Na/K (+) 14.94%	(Xiong et al., 2025)
Antioxidant response	Cluster bean (*Cyamopsis tetragonoloba* L.)	Pot experiment; salinity stress (0, 6, and 12 dS m^−1^) silicon dioxide nanopowder (50 mg L^−1^)	POD (+) 48.9-62.6% SOD (+) 76-86.7% CAT (+) 89.1-92.7% APX (+) 49.6-32% phenolic (+) 10.1-55.08% flavonoid (+) 13.8-16.6% MDA (-) 52.9-57.2% H_2_O_2_ (-) 51.4-59.1%	(Rahimi et al., 2026)
	Cucumber (*Cucumis sativus* L., cv. *9930*)	Hydroponic experiment; control (CK), 100 mM NaCl (Na), 100 mM NaCl + 1.5 mM Na_3_SiO_3_ (Na+Si1), 100 mM NaCl + 3 mM Na_3_SiO_3_ (Na+Si2), 100 mM NaCl + 1.5 mM SiNPs (Na+SiNPs1), and 100 mM NaCl + 3 mM SiNPs (Na+SiNPs2).	Shoot: MDA (-) 12.34-15.62% REL (-) 1.72-9.60% SOD (+) 21.54-94.62% CAT (+) 18.89-23.96% APX (+) 55.29-74.05% GPOD (+) 62.01-133% GR (+) 72.16-109.98% Root: MDA (-) 6-29.30% H_2_O_2_ (-) 35.00-49.84%	(Dong et al., 2026)
	Chia (*Salvia hispanica* L.)	Pot experiment; 0, 50, and 100 mM NaCl, 0, 100, 200, and 400 mgL^−1^ SiNPs	Proline (+) 90.1% Carbohydrates (+) 146% Flavonoids (+) 78% phenols (+) 83.4% SOD (+) 147%% CAT (+) 183% APX (+) 334%	(Haghaninia et al., 2025)
	maize (*Zea mays* L.)	Pot experiment; 60 and 120 mM NaCl 2, 4, 6–8 mM Si (Na_2_SiO_3_);	phenolics (+) 6.08-17.03% flavonoids (+) 2.14-7.50% H_2_O_2_ (-) 4.61-24.06% SOD (+) 12.78-50.57% POD (+) 5.06-15.58% CAT (+) 0.85-10.06%	(Ullah et al., 2025)
Hormonal signaling	Rice (*Oryza sativa* L.)	Hydroponic experiment; 2 mmol L^-1^ nano-silica, 60 mmol L^-1^ NaCl	Leaves: IAA (+) 22.08% CTK (+) 30.87% GA (+) 36.70% ABA (+) 15.45% SA (+) 32.17% JA (+) 22.21% Roots: IAA (+) 56.14% GA (+) 2.65% ABA (+) 13.23% SA (+) 27.71% JA (+) 24.62%	(Xiong et al., 2025)
	Licorice (*Glycyrrhiza uralensis*)	Sand culture experiment; 100 mM NaCl; 2- and 3-mM Si (K_2_SiO_3_)	After 10 days of treatment GA_3_ (+) 37.67-54.54% IAA (+) 82.35-129.41% ABA (+) 112.5% After 20 days of treatment IAA (+) 54.41-82.35%; ABA (+) 94.64%	(Bokor et al., 2019)
	Soybean (*Glycine max* L. cv. *Taekwangkong*)	Hydroponic experiment; 80 mM NaCl; 2.5 mM Na_2_SiO_3_	GA_4_ (+) 48.02% ABA (-)	(Lee et al., 2010)
	Grapevine (*Vitis vinifera* L. cv. *castellana negra*)	Pot experiment; 120 mM NaCl, 150, 200 and 250 mg L^-1^ SiNPs	ABA (-) 38-61% IAA (+) 88-142% CTK (+) 47-61% BR (+) 65-85% ET (+) 96-205%	(Khan et al., 2024)
Gene regulation	Tomato (*Solanum lycopersicum* ‘Dongnong708’)	Pot experiment; 150 mM NaCl with or without 2 mM sodium silicate	Upregulated: ET biosynthesis process genes (solyc02g091990.3, Solyc01g095080.3, and Solyc02g063541.1), two ABA biosynthesis genes (Solyc07g056570.1 and Solyc08g016720.1), one IAA-related gene (Solyc06g075690.3), and one CTK-related gene (Solyc01g107730.3), three xyloglucan endotransglucosylase/hydrolase genes (Solyc03g093080.3, Solyc12g017240.2, and Solyc03g093130.3), four genes (Solyc06g076330.3, Solyc09g010990.3, Solyc09g014240.3, and Solyc10g076830.2) encoding laccase.	(Wang et al., 2026d)
	Cotton (*Gossypium hirsutum* L. cv. Zhong0102)	Hydroponic experiment; 150 mM NaCl; 0.4 mM Si (Na_2_SiO_3_·9H_2_O)	GhNCED (U) GhCYP707A (D) Sixteen genes related to water transport were upregulated, including nine *PIPs*, five *TIPs*, one *NIP*, and one *SIP* homologs.	(Wang et al., 2026b)
	Maize (*Zea mays* L.)	Hydroponic experiment; 40 mM NaCl; 1.5 mM Si(OH)_4_	ZmSOS1 (U), ZmSOS2 (U), ZmHKT1 (D)	(Bosnic et al., 2018)
	Cucumber (*Cucumis sativus* L. ‘Jinyou 1’)	Hydroponic experiment; 75 mM NaCl, 0.3 mM Si (Na_2_SiO_3_·9H_2_O)	RAP2 (U), MYB44-like (U), NAC 69-like (D), bHLH47-like (D), cationic amino acid transporter (Csa1G056920) (D), MAPKKK (Csa1G532310) (U), serine/threonine-protein kinase (Csa2G354110) (U); oxidative stress protein (Csa2G000790, Csa3G435530) (U), auxin-induced protein (Csa6G104650)	(Zhu et al., 2019a)
	Cucumber (*Cucumis sativus* L. ‘Jinyou 1’)	Petri dishes; CK, distilled water; 200 mM NaCl; 200 mM NaCl + 0.3 mM Si (as sodium silicate)	12 h: NCED2 (U) CYP707A1 (U) GA20ox (U) GA3ox (U) 36 h: NCED1 (D) NCED2 (D) CYP707A1 (D) GA20ox (D) GA3ox (D) GA2ox (D)	(Gou et al., 2020)
Microbial interaction	Soybean (*Glycine max* L.)	Pot experiment; 8-10 °C, 1.0 mM H_2_SiO_4_	*Proteobacteria* (+) *Bacteroidota* (+) *Ascomycota* (+) *Edaphobacter* (+) *Haliangium* (+) *Streptomyces* (+)	(Ahmad et al., 2024a)
	sugarcane	Field plot experiment; CK, compound fertilizer; silicon fertilizer	Root: *Acidiphilium* (+) *Fusobacterium* (+) *Allobaculum* (+) Chujaibacter (-) Opitutus (-) Amycolatopsis (-) Haliangium(-) Bauldia (-) Saccharothrix(-) Rhizosphere: Mesorhizobium (+) Sinomonas (+) Enterobacter (+) Olivibacter (+) Glycomyces (+) Mucispirillum (+) Methylopila (+) Ochrobactrum (-) Amycolatopsis (-) Bacillus (-) Haematomicrobium (-) Alcaligenes (-) Aquicella (-) Corynebacterium_1 (-) Acinetobacter (-) Brachybacterium (-) Lactobacillus (-) Leucobacter (-) Pseudomonas (-) Aeromonas (-) Brevibacterium (-) Akkermansia (-) Arthrobacter (-)	(Yuan et al., 2022)
	Soybean (*Glycine max* L.)	Pot experiment; Control (nonsalt stress); 200 mM NaCl; SiO_2_-COOH (SiO_2_-COOH NPs + salt stress)	L-tyrosine (+), uridine (+), indole-3-acetamide (+), Variovorax (+), Pseudomonas (+), Haliangium (+), Arthrobacter (+)	(Chen et al., 2025)
	Soybean (*Glycine max* L.)	Pot experiment; 100 mM NaCl; SiO_2_ NPs+100 mM NaCl	Pseudomonas (+), Bacillus (+), Variovorax I (+)	(Wang et al., 2024a)

H_2_O_2_, hydrogen peroxide; MDA, malondialdehyde; REL, relative electrolyte leakage; GPOD, guaiacol peroxidase; GR, glutathione reductase; Si NPs, silicon nanoparticles; SOD, superoxide dismutase; APX, ascorbate peroxidase; CAT, catalase; IAA, indole-3-acetic acid; GA, gibberellin; CK, cytokinin; BR, brassinosteroid; ABA, abscisic acid; ET, ethylene. U, upregulation; D, downregulation; Positive (+) and negative (−) values indicate increases and decreases, respectively, following Si application under salt stress. Percentage change was calculated as [(Si-treated value − corresponding non-Si value)/corresponding non-Si value] × 100%.

## 5 Context dependence and broader cross-tolerance of Si-mediated stress alleviation

### 5.1 Boundary conditions, neutral responses, and species specificity of Si-mediated stress alleviation

Although Si application has frequently been associated with improved plant performance under salinity and other environmental stresses, its effects are not universal. The available literature includes complete or partial non-responses, endpoint-specific neutral effects, declining efficacy under severe stress, genotype-dependent responses, and adverse effects caused by excessive Si supply. These contrasting outcomes indicate that Si should be regarded as a context-dependent stress-mitigation treatment rather than a universally effective regulator. The magnitude and direction of the response depend on plant species and genotype, inherent Si-accumulation capacity, developmental stage, stress intensity and duration, Si source and concentration, application route, and the physicochemical properties of the growth medium.

Species differences in Si uptake and accumulation constitute an important source of response heterogeneity. Si accumulation varies substantially across plant phylogeny, with many species in Poales accumulating relatively high shoot Si concentrations, whereas numerous eudicots accumulate considerably less Si. These differences are related to root uptake capacity, transporter expression and localization, transpiration, and long-distance Si distribution (Hodson et al., 2005; Ma and Yamaji, 2006). High Si accumulation may favor extensive deposition in roots, cell walls, and aerial tissues and may therefore enhance structural or apoplastic protection. Nevertheless, accumulation type should not be treated as a deterministic predictor of stress alleviation. Low-accumulating species, including tomato and lettuce, can respond positively to Si through improved water relations, local membrane or root effects, foliar uptake, or rhizosphere modification. Conversely, even high-accumulating grasses do not exhibit positive responses in every organ, trait, genotype, or treatment condition. Thus, the relevant distinction is not simply between Si accumulators and non-accumulators, but between combinations of uptake capacity, Si delivery, tissue availability, and the mechanism limiting plant performance under a particular stress.

Several salinity studies have documented endpoint-specific neutral responses despite improvements in other traits. In tomato, Si supplementation under 80 mM NaCl improved biomass, tissue water content, leaf turgor, and photosynthetic performance, but it did not significantly alter total salt entry into the plant, the distribution of salt among organs, or whole-plant water uptake. The beneficial response was therefore attributed primarily to improved tissue water storage and salt dilution rather than to reduced ion uptake (Romero-Aranda et al., 2006). In wheat, Si increased shoot dry weight under part of the salinity treatment, whereas root dry weight and chlorophyll content remained unaffected (Ahmad et al., 1992). Similarly, in three rice cultivars, Si improved shoot growth and reduced shoot Cl^-^ concentration under salinity, but root growth and root Cl^-^ concentration remained unchanged, and shoot relative growth rate was not consistently improved (Shi et al., 2013). These findings demonstrate that a positive response in one physiological endpoint should not be generalized to the entire plant or interpreted as evidence that all commonly proposed Si-mediated mechanisms were activated.

The distinction between vegetative improvement and protection of agronomically important endpoints is also important. In greenhouse rose, increased Si supply enhanced vegetative growth and improved plant appearance under both low- and high-salinity conditions. However, Si was unable to alleviate the adverse effects of NaCl on flower production and flower quality, and net photosynthesis, stomatal conductance, and transpiration were unaffected by Si. Thus, improvement in vegetative biomass did not fully protect the economically important reproductive product (Savvas et al., 2007). Likewise, the co-application of Si and a six-strain *Bacillus* inoculum in salt-stressed cucumber did not produce a detectable interactive effect on plant growth, even though Si alone and the bacterial treatment individually improved some traits. Neither treatment affected growth under non-stress conditions (Kaloterakis et al., 2021). This result is particularly relevant to claims of Si–microbe synergy: the superior performance of a combined treatment should not be assumed unless it is supported by a statistically significant interaction or an appropriate additivity model.

Si responsiveness may also vary markedly among genotypes within the same species. In two maize cultivars exposed to 60 mM NaCl, increasing Si concentrations produced predominantly positive responses in ‘Kosmo 230’, whereas some growth parameters were depressed in ‘SMH 220’, particularly at the highest Si supply. Chlorophyll content increased under stress, but chlorophyll-fluorescence parameters remained unchanged. These results demonstrate that even closely related genotypes may differ in their effective Si-dose range and in the physiological processes that respond to Si (Sacała, 2017). Genotype-dependent responses may reflect variation in Si uptake and transport, baseline stress tolerance, ion selectivity, root architecture, or the capacity to use Si-induced changes in antioxidant and osmotic regulation. Consequently, treatment recommendations derived from a single genotype should not be generalized to an entire crop species.

The effectiveness of Si also appears to operate within dose- and stress-dependent windows. In GF677 (*Prunus dulcis* × *Prunus persica*) and GN15 (*Prunus dulcis* × *Prunus persica*) rootstocks, Si had no noticeable effect on leaf or root Cl^-^ accumulation under 50 mM NaCl. Under 100 mM NaCl, 2 mM Si significantly reduced Cl^-^ accumulation, whereas 1 mM Si produced only a slight and non-significant reduction in several Cl^--^related endpoints. The same experiment showed that Si had little or no effect on root biomass and stomatal conductance under non-saline conditions, although it improved several traits under salinity (Gharbi et al., 2025). These results indicate that an ineffective treatment may reflect insufficient Si availability relative to the intensity or mechanism of stress rather than an absolute absence of Si responsiveness. At the opposite end of the response range, the protective efficacy of Si in Juniperus excelsa declined under the highest salinity treatment, showing that Si could not fully compensate for injury once stress severity exceeded the effective protective range of the treatment (Yucedag and Gailing, 2026).

Neutral or adverse responses may also occur when Si is supplied in the absence of substantial stress or at excessive concentrations. In dill, sodium silicate improved dry matter, ion balance, and antioxidant responses under saline conditions but negatively affected growth under non-saline conditions, indicating that the cost–benefit balance of Si depended on the environmental context (Shekari et al., 2017). A recent lettuce study provided a particularly clear dose-response example. Under 100 mM NaCl, foliar application of 3.66 mM SiO_2_ produced the strongest improvements in fresh weight, plant diameter, chlorophyll content, and anthocyanin content. Increasing the concentration above this optimum progressively reduced the benefits, while 29.30 mM SiO_2_ caused reduced growth, marked leaf chlorosis, and early necrosis. Moreover, the otherwise effective 3.66 mM treatment reduced lettuce fresh weight under severe drought, demonstrating that a dose beneficial under salinity may become neutral or detrimental under a different stress condition (Simko et al., 2025).

Publication and reporting practices may further contribute to an overly positive view of Si effects. Experiments commonly measure many physiological, biochemical, and molecular variables, but positive results may receive greater emphasis than unchanged or adverse endpoints. Future studies should report complete response profiles and distinguish prespecified primary outcomes from exploratory variables. Factorial designs should explicitly test genotype × Si dose × formulation × application route × stress-intensity interactions, while multilevel or dose-response models should be preferred over comparisons involving a single Si concentration. Greater emphasis is also required on reproductive stages, product quality, yield, multiseason field performance, and gradual soil salinization rather than short-term seedling responses alone.

Overall, current evidence supports a conditional rather than universal model of Si-mediated stress alleviation. High Si accumulation may facilitate efficient uptake, transport, and structural deposition, but it does not guarantee improvement in every trait or under every environmental condition. Low-accumulating species may still respond when Si is supplied in an accessible form or through an appropriate application route. The central question is therefore not simply whether Si improves stress tolerance, but under which biological and environmental conditions, at what dose, through which mechanism, and for which agronomically meaningful endpoint it is effective.

### 5.2 Evidence for broader Si-associated stress protection

Evidence from multiple plant systems indicates that Si treatment can be associated with improved responses to salinity, drought, heat, metal toxicity, and pathogen challenge. However, the magnitude and reproducibility of these effects vary substantially among plant species, genotypes, developmental stages, Si formulations, application routes, doses, and stress conditions. In this review, broader stress protection refers to the observation that Si treatment has been linked to beneficial responses across different stress systems. This concept should be distinguished from cross-tolerance in the strict sense, which requires evidence that an acclimated state induced by Si or by one stress subsequently enhances tolerance to a different stress. Most available studies demonstrate protection against individual stress types rather than experimentally verified transfer of tolerance between sequential or heterologous stresses.

Under abiotic stress, Si-associated protection frequently involves physiological processes that are also recruited during salinity responses. Under salt stress, Si treatment has been reported to improve K^+^/Na^+^ homeostasis, limit oxidative injury, and support photosynthetic performance and growth (Meng et al., 2020; Etesami et al., 2022; Peña Calzada et al., 2023; Xiong et al., 2025). Under drought, Si application has been associated with improved plant water status, water-use efficiency, osmolyte accumulation, membrane stability, and antioxidant regulation (Saja-Garbarz et al., 2021; Johnson et al., 2022). During heat stress, Si-treated plants may maintain photosynthetic function and membrane integrity and exhibit altered accumulation of heat-shock proteins and other molecular chaperones (Khan et al., 2020; Chaganti et al., 2023). Although these responses involve overlapping protective processes, such as redox control, osmotic adjustment, and stabilization of cellular structures, their relative importance depends on the dominant injury mechanism in each stress environment.

Si treatment has also been associated with enhanced resistance to several fungal and bacterial pathogens. Proposed mechanisms include increased silicification and cell-wall reinforcement, greater accumulation of phenolic and antimicrobial compounds, altered expression of pathogenesis-related proteins, and modulation of SA- and JA-associated defense responses (Vivancos et al., 2016; de Souza Alonso et al., 2022; Chen et al., 2024; Ji et al., 2025). Nevertheless, the contribution of physical and inducible biochemical defenses differs among plant–pathogen systems, and Si-mediated disease suppression should not be assumed to operate through a single conserved mechanism. Similarly, under cadmium, arsenic, aluminum, and other metal stresses, Si has been reported to reduce metal uptake or root-to-shoot translocation, promote sequestration or co-deposition, stabilize membranes and cell walls, and support antioxidant and detoxification systems (Bhat et al., 2019). These processes may reduce cellular injury, but their effectiveness depends strongly on plant genotype, metal species, soil chemistry, Si source, and treatment concentration.

Taken together, the available evidence suggests that Si treatment can influence several protective processes that are repeatedly recruited under otherwise distinct stresses, including ion regulation, osmotic adjustment, redox homeostasis, hormonal responses, defense-related metabolism, and structural reinforcement. However, the coexistence of these responses across separate experiments does not by itself establish a stable or transferable primed state. Direct evidence for true Si-induced cross-tolerance remains limited because relatively few studies have employed sequential stress designs, recovery periods, heterologous challenge experiments, or measurements of persistent stress memory. Broader Si-associated stress protection should therefore be interpreted as a context-dependent phenomenon that may arise from partial reuse of common defense modules rather than as a universal systemic program.

### 5.3 A proposed shared defense network underlying broader Si-associated stress protection

A plausible explanation for the reported effects of Si across different stress systems is that Si treatment influences defense modules that are repeatedly recruited during salinity, drought, heat, metal toxicity, and pathogen challenge. These modules include second-messenger and hormonal responses, protein-kinase-dependent signal decoding, transcriptional regulation, antioxidant and detoxification systems, osmotic protection, and structural reinforcement. Rather than constituting a single linear pathway, they form an interconnected network in which changes in ionic, redox, and water homeostasis can alter the activation threshold and efficiency of pre-existing stress-response mechanisms. This framework should be regarded as a proposed integration of currently fragmented evidence rather than as a fully established Si-specific signaling pathway.

At the signaling level, Si-associated improvements in membrane stability, ion balance, ROS status, and tissue water relations may influence Ca^2+^-, NO-, ABA-, JA-, and SA-related responses. In canonical plant stress pathways, Ca^2+^ signals are decoded by calmodulin-related proteins, CDPKs, and CBL–CIPK complexes, whereas ABA and other hormones engage SnRK2-, MAPK-, and receptor-dependent signaling modules. These pathways converge on transcription factors such as NAC, WRKY, MYB, bZIP, and HSF, which regulate stress-responsive genes involved in ion transport, redox regulation, osmotic adjustment, protein protection, and defense metabolism. In several experimental systems, Si treatment has been associated with altered expression of NAC-, WRKY-, MYB-, and HSF-family transcription factors (Mittal et al., 2009; Khattab et al., 2014; Farooq et al., 2016; Maghsoudi et al., 2018; Jin et al., 2024), together with changes in pathogenesis-related proteins, including chitinases and glucanases, and in protective proteins such as LEA and heat-shock proteins (Dann and Muir, 2002; Farooq et al., 2009; Dorneles et al., 2017; Fischer et al., 2024; Lata-Tenesaca et al., 2024). However, most kinase-to-transcription-factor connections have been established in general plant stress signaling, whereas direct evidence that Si modifies the activity, phosphorylation state, DNA-binding capacity, or temporal dynamics of these components remains limited.

A second shared module involves redox regulation and cellular detoxification. Si treatment has frequently been associated with altered activities of SOD, CAT, APX, GR, and other antioxidant enzymes, together with regulation of the ascorbate–glutathione cycle and reductions in excessive ROS accumulation, lipid peroxidation, and electrolyte leakage (Das et al., 2018; Ahmad et al., 2019; Singh et al., 2022). These responses may provide broadly relevant protection because oxidative damage is a common secondary consequence of salinity, drought, heat, metal toxicity, and pathogen attack. Nevertheless, increased antioxidant-enzyme activity should not automatically be interpreted as direct activation by Si or as proof of stronger tolerance. Enzyme activities may increase because defense is activated, remain unchanged because oxidative pressure has already been reduced, or decline during later recovery. Their interpretation therefore requires explicit consideration of stress intensity, sampling time, tissue, and accompanying damage and growth endpoints.

Structural reinforcement represents another recurring component of Si-associated protection. Si deposition in cell walls, epidermal tissues, and apoplastic barriers can increase tissue rigidity, reduce uncontrolled water loss, restrict the movement of toxic ions and metals, and impede pathogen penetration (Kim et al., 2002; Hattori et al., 2003; Huang et al., 2024). Si-associated membrane stabilization may additionally help preserve ion gradients, water transport, receptor function, and intracellular signaling under stress. These structural effects are supported by relatively substantial anatomical and physiological evidence, but they do not occur uniformly in all species because Si uptake, transport, and deposition vary markedly between high-, intermediate-, and low-accumulating plants. Structural protection should therefore be considered one component of the proposed network rather than a universal explanation for all Si responses.

Overall, the proposed shared defense network provides a mechanistic basis for understanding why Si treatment may improve performance under multiple stress conditions. The strongest evidence currently supports Si-associated changes in structural protection, ionic and redox homeostasis, antioxidant regulation, and selected stress-responsive genes. By contrast, direct evidence for Si-dependent modulation of Ca^2+^ and NO signal kinetics, kinase activation, transcription-factor binding, long-term priming, and stable stress memory remains insufficient. Si should therefore be viewed as a potential modulator of pre-existing defense systems whose effectiveness depends on biological and environmental context, rather than as a universal signal that directly activates every component of a common stress-response network.

## 6 Formulation-dependent Si-based strategies: conventional Si fertilizers versus nano-Si formulations

### 6.1 Conventional Si fertilizers: agronomic reliability and field scalability

Conventional Si fertilizers, including calcium silicate, potassium silicate, sodium silicate, magnesium silicate, wollastonite, slag-based silicates, silicate minerals, and stabilized silicic acid products, remain the most established route for agricultural Si supplementation (Bhat et al., 2021). Their major advantage lies in agronomic reliability: they are generally inexpensive, available at large scale, compatible with existing fertilization systems, and suitable for broad-acre field application. In Si-accumulating and intermediate Si-accumulating crops such as rice, sugarcane, wheat, and maize, conventional Si fertilization can enhance tissue silicification, strengthen cell-wall and epidermal barriers, and reduce stress-induced physiological injury (Lei et al., 2025). These protective effects are commonly associated with improved water status, stimulation of antioxidant systems, regulation of ion homeostasis, reduced uptake or translocation of toxic metals, and enhanced structural resistance to pathogens and herbivores (Laifa et al., 2024; Priya and Kumar, 2024; Shang et al., 2025; Dong et al., 2026).

A key advantage of conventional Si fertilizers is that they can provide residual soil-mediated effects in addition to direct plant physiological responses. Soil-applied silicates can simultaneously supply Si, modify rhizosphere chemistry, contribute accompanying cations such as Ca^2+^, K^+^, or Mg^2+^, and influence nutrient availability (Ono et al., 2025; Teixeira et al., 2025). This is particularly relevant in acidic, saline, degraded, or metal-contaminated soils, where Si amendments may reduce metal bioavailability, improve nutrient balance, and modify interactions among Si, phosphorus, calcium, magnesium, aluminum, and trace metals.

Despite these advantages, conventional Si fertilizers have clear limitations. Many mineral or slag-based Si sources dissolve slowly, and the release of plant-available monosilicic acid is strongly affected by soil pH, organic matter, ionic strength, salinity, calcium and magnesium concentrations, and adsorption reactions with soil colloids (Babu et al., 2016; Anjum and Prakash, 2026). The influence of pH is especially complex. Haynes (2019) emphasized that liming may increase Si availability in the short term by stimulating dissolution of biogenic Si, but may reduce soluble Si in the longer term by promoting Si adsorption or precipitation processes. Thus, soil pH does not exert a uniform effect on Si bioavailability, and the same Si source may perform differently across soil types. Moreover, the agronomic performance of conventional Si fertilizers is not determined by total Si concentration alone. Keeping (2017) showed in sugarcane that the total Si content of applied sources did not necessarily predict plant-available soil Si or subsequent leaf Si accumulation, indicating that mineralogy, dissolution behavior, and soil chemistry are more important than total Si content alone.

Another limitation is that repeated or high-dose application of conventional Si fertilizers may alter soil ionic balance. Depending on the source, conventional Si amendments may introduce Ca, Mg, K, or Na, shift pH, influence phosphorus sorption, and alter competitive adsorption among major cations (Haynes and Zhou, 2018; Schaller et al., 2019; Uhuegbue et al., 2024). These changes may be beneficial in acidic or degraded soils, but they may also be undesirable in saline-sodic, calcareous, or nutrient-imbalanced systems. Therefore, conventional Si fertilizers should be considered soil-integrated stress-buffering tools rather than universally neutral Si supplements. Their field value depends on matching fertilizer source, soil chemical background, crop Si demand, and long-term nutrient management.

### 6.2 Nano-Si formulations: enhanced reactivity but uncertain ecological fate

Nano-Si formulations, most commonly Si nanoparticles or silica nanoparticles, have emerged as promising materials for improving Si delivery and stress tolerance. Their potential advantages arise from nanoscale size, high surface-area-to-volume ratio, high surface reactivity, tunable surface charge, and possible enhancement of plant uptake efficiency (Bhat et al., 2021; Shilpi et al., 2021). Depending on particle size, surface chemistry, and dissolution behavior, nano-Si formulations may interact with root or leaf surfaces, partially dissolve into silicic acid, or enter plant tissues through apoplastic and possibly symplastic routes. However, the extent to which intact nanoparticles are absorbed and translocated remains unresolved (Bhat et al., 2021). Compared with bulk Si sources, Si nanoparticles may interact more efficiently with root surfaces, leaf epidermis, stomatal openings, cell-wall pores, and apoplastic pathways (Pan et al., 2023; Djanaguiraman et al., 2024). They may also partially dissolve into silicic acid and subsequently enter conventional Si uptake pathways, suggesting that their biological effects may involve both nanoparticle-specific behavior and classical Si nutrition (Bhat et al., 2021; Mukarram et al., 2022; Pan et al., 2023).

Under stress conditions, nano-Si treatment has been associated with changes in plant morphological, physiological, biochemical, and molecular responses. Reported effects include improved seed germination (Wang et al., 2024b), enhanced root growth (Sayed et al., 2022), increased chlorophyll content (Wang et al., 2025), improved photosynthetic performance (Shoukat et al., 2025b), higher relative water content (Badawy et al., 2021), stronger antioxidant defense (Rahimi et al., 2026), lower accumulation of reactive oxygen species and malondialdehyde, improved K^+^/Na^+^ balance (Xiong et al., 2025), reduced uptake or translocation of toxic metals (Shoukat et al., 2025a), and modulation of stress-responsive genes (Wang et al., 2024b, 2025). These effects make nano-Si particularly attractive for seed priming, foliar spraying, hydroponic systems, protected cultivation, and high-value horticultural crops, where low-dose and rapid-response interventions may be more practical than large-scale soil amendment.

Several direct comparative studies indicate that nano-Si can outperform conventional Si sources in specific stress contexts. Wang et al. (2024b) showed that Si nanoparticles improved tomato seed germination under salt stress more effectively than conventional Si, increasing germination percentage, fresh weight, and Si accumulation while more strongly regulating antioxidant enzymes and gibberellin/abscisic acid metabolism-related genes. Morshedloo et al. (2025) investigated ginger mint (*Mentha gracilis R.Br.*) and demonstrated that foliar application of both conventional Si and nano-Si alleviated the adverse effects of drought stress on plant growth and physiological performance. Notably, the application of 50 mg L^-^¹ nano-Si resulted in the highest biomass accumulation in ginger mint plants. Compared with conventional Si, nano-Si is considered more effective in some experimental systems because of its larger specific surface area, which may increase its solubility, reactivity, and interaction with biological structures (Gao et al., 2022). Rizwan et al. (2023) compared Si nanoparticles with conventional Si amendments in maize grown in saline-sodic soil and found that nano-Si and potassium silicate improved plant growth, nutrient availability, physiological traits, and salt-stress mitigation, although the relative advantage depended on crop season and residual effects. Specifically, nano-Si exerted a stronger growth-promoting effect during the first growing season, while potassium silicate showed a more evident residual benefit in the subsequent growing season. These findings support the view that nano-Si may offer stronger short-term physiological stimulation, whereas some conventional Si fertilizers may retain greater soil residual value. In addition, nano-Si application promoted cucumber biomass accumulation under salt stress more effectively than silicate treatment and induced broader changes in gene expression networks. Several key target genes involved in nano-Si-mediated regulation of salt-stress responses in cucumber were also identified (Dong et al., 2026). In that cucumber system, nano-Si treatment was associated with broader transcriptomic changes than the tested conventional silicate treatment.

Nevertheless, the agronomic application of nano-Si remains constrained by formulation-dependent uncertainty. The behavior of Si nanoparticles is highly sensitive to particle size, surface charge, coating, aggregation, dissolution kinetics, soil pH, ionic strength, organic matter, and microbial interactions. Therefore, different materials labeled as “SiNPs” may exhibit distinct plant availability, biological activity, mobility, persistence, and ecological effects. Although soluble Si uptake and transport have been relatively well characterized, the absorption, translocation, and subcellular fate of Si nanoparticles remain poorly understood (Yan et al., 2024). These uncertainties limit the direct extrapolation of controlled experimental results to field-scale agricultural application.

### 6.3 Field translation, economic feasibility, environmental safety, and decision framework

A critical perspective is needed when comparing conventional and nano-Si strategies. Nano-Si should not be regarded as inherently more advanced or universally superior, because its benefits are highly context- and formulation-dependent. Although recent reviews have highlighted the potential of SiNPs to improve plant growth and stress tolerance, they also emphasize that SiNP performance depends strongly on particle properties, plant species, application route and environmental conditions (Bhat et al., 2021; Pan et al., 2023; Yan et al., 2024). Therefore, the apparent advantages of nano-Si must be evaluated alongside uncertainties in formulation stability, aggregation, dissolution kinetics, soil retention, mobility, crop safety, microbial effects and long-term ecological fate.

The ecological fate of nano-Si remains a major knowledge gap. In real soils, nanoparticles may undergo homoaggregation, heteroaggregation with clay minerals or organic matter, dissolution, sorption, surface transformation, and biological modification. These processes determine whether nano-Si remains bioavailable, becomes immobilized near the application zone, transforms into soluble Si species, or migrates into non-target soil and water compartments. Although silica-based nanoparticles are often considered less hazardous than many metal-based nanoparticles, their agricultural safety cannot be assumed without formulation-specific assessment. Nano-Si may alter soil physicochemical properties, microbial biomass and diversity, enzyme activity, nutrient cycling, respiration, and plant–microbe interactions, and these outcomes depend on both the original material and its transformation trajectory in the soil–plant system (Pan et al., 2023; Wei et al., 2024; Zhang et al., 2024a; Haydar et al., 2025). Nano-Si safety should therefore be evaluated at the soil–plant–microbiome interface rather than inferred solely from short-term plant growth responses.

Another key limitation is the lack of standardized comparisons between conventional and nano-Si formulations. Many nano-Si studies are still based on germination, hydroponic, pot or short-term stress experiments, whereas long-term, multi-season and multi-soil field trials remain scarce. Differences in Si dose, application route, pH, particle characterization, stress severity, genotype and growth stage further limit cross-study comparability. Without equal-Si-dose, equal-pH and comparable application-route controls, it remains difficult to distinguish whether the observed benefits of nano-Si arise from nanoscale behavior, higher Si availability, altered pH, surface reactivity or delivery-mode effects. Recent salinity-focused reviews also note that comparative studies among different Si forms remain limited, even though both Si nanoparticles and conventional Si compounds have shown potential for improving stress tolerance (Siddiqi et al., 2025).

Agronomic efficacy alone is insufficient to determine whether a Si intervention is economically viable. Field-scale adoption depends on the cost of the Si source, transportation, storage, application equipment, labor, application frequency, and the magnitude and stability of the resulting yield or quality benefit. A cost–benefit analysis suggested that Si fertilization may be economically feasible in many agronomic settings, although affordability may remain a constraint for smallholder farmers and will vary with crop value, expected yield gain, fertilizer source, and production costs (Thorne et al., 2020). Conventional silicate fertilizers may be more compatible with broad-acre production because several sources can be applied through established soil-fertilization practices and may provide residual effects on soil Si availability. However, the agronomic value of a product cannot be predicted from its total Si content alone, because mineralogy, solubility, liming capacity, and soil pH strongly influence plant-available Si and crop uptake (Keeping, 2017). Field-based evidence in wheat further indicates that Si supplementation can protect biomass, grain production, and water-use efficiency under water limitation, but such biological benefits still need to be evaluated against input and application costs under commercial production conditions (Johnson et al., 2022). Nano-Si formulations may permit lower application rates and targeted delivery through seed priming or foliar application, but economic assessments should also account for material synthesis, surface modification, characterization, formulation stabilization, regulatory evaluation, and specialized application requirements. Future field studies should therefore include partial-budget analysis, benefit–cost ratios, marginal returns, and risk-adjusted profitability across multiple seasons rather than reporting biological efficacy alone. Economic recommendations should also account for crop market value, baseline soil Si availability, stress frequency, irrigation-water quality, and possible residual benefits to soils or subsequent crops.

Long-term sustainability should be evaluated across the complete soil–plant–management system rather than inferred from short-term improvements in plant growth. Conventional silicate sources can modify soil pH, plant-available Si, and nutrient availability, but these effects vary substantially with source mineralogy, solubility, soil properties, and application rate (Keeping, 2017). Calcium silicate application has also been associated with trade-offs among nutrient-use efficiency, crop biomass, grain yield, and greenhouse-gas emissions, indicating that improvement in one environmental or agronomic endpoint does not necessarily represent an overall sustainability benefit (Ku et al., 2020). Repeated applications should therefore be evaluated for changes in soil pH, phosphorus availability, nutrient balance, and inputs of accompanying cations such as Na^+^, K^+^, Ca^2+^, and Mg^2+^. For nano-Si formulations, the transformation, mobility, and rhizosphere effects described above require long-term, formulation-specific monitoring under realistic field conditions.

Multi-season studies should therefore quantify residual plant-available Si, changes in soil chemical properties, nutrient-use efficiency, microbial community stability, soil enzyme activity, soil-fauna responses, leaching, and possible transfer into non-target environmental compartments. Life-cycle assessments should additionally consider energy use, raw-material sourcing, formulation production, transportation, application frequency, and end-of-life environmental costs. The most sustainable strategy may not be the formulation producing the largest short-term physiological response, but the treatment that achieves reliable yield protection at the lowest effective dose, with acceptable economic returns, limited ecological disturbance, and manageable residual effects.

Based on current evidence, field application should follow a scenario-specific decision framework. Conventional Si fertilizers may be better suited to broad-acre systems where cost, residual soil effects, and field scalability are priorities. Nano-Si formulations may warrant evaluation for low-dose seed or foliar delivery in protected cultivation and high-value crops, but their suitability should be established for each formulation, crop, soil, and stress scenario.

Future research should move beyond simple efficacy comparisons and adopt standardized, mechanism-oriented and risk-aware designs. Direct comparisons should use equal Si doses, equal pH, comparable application routes, realistic soil conditions and well-characterized materials. Nano-Si formulations should be characterized for particle size, hydrodynamic diameter, zeta potential, morphology, crystallinity, surface coating, aggregation behavior, dissolution kinetics and purity. Field trials should further include multiple seasons, soil types, dose–response gradients, crop yield and quality, plant Si accumulation, microbial community structure, soil enzyme activity, soil fauna responses, residual behavior and leaching potential. Only through such an integrated framework can nano-Si be positioned scientifically—not as a universal replacement for conventional Si fertilizers, but as a formulation-specific option with potential efficiency advantages, ecological risks, and corresponding stewardship requirements.

## 7 Conclusions and future perspectives

### 7.1 Mechanistic synthesis: a context-dependent inside–outside model of Si-associated salt tolerance

The principal mechanistic insight emerging from this review is that Si-associated salt tolerance is unlikely to result from the activation of a single receptor, signaling pathway, transporter, or antioxidant system. Instead, Si appears to modify the physicochemical and biological conditions under which pre-existing plant stress-response networks operate. Lsi-family transporters determine Si uptake and long-distance distribution, whereas tissue-specific Si deposition at the root endodermis, cell walls, vascular interfaces, membranes, and aerial tissues establishes the spatial basis for its effects. By reinforcing apoplastic barriers, stabilizing plasma membrane and tonoplast integrity, restricting excessive Na^+^ entry and root-to-shoot transport, preserving K^+^ retention, and limiting uncontrolled oxidative injury, Si creates a more stable intracellular environment for stress perception, signal transmission, and metabolic acclimation. Thus, structural protection, ionic regulation, and redox buffering should not be viewed as independent Si effects, but as interconnected processes that establish the biophysical foundation for subsequent signaling responses.

Within this stabilized cellular environment, Si treatment is associated with dynamic changes in phytohormone and second-messenger systems. The available evidence does not support a simple model in which Si consistently activates or suppresses ABA, JA, SA, cytokinin, ethylene, Ca^2+^, or NO signaling. Instead, Si-associated responses vary with tissue, developmental stage, stress intensity, and sampling time. Early increases in ABA or antioxidant activity may support stomatal regulation and rapid acclimation, whereas later normalization of hormone concentrations or antioxidant-enzyme activities may reflect reduced stress pressure and recovery of homeostasis. Similarly, increased nitrate-reductase activity and reduced S-nitrosothiol accumulation may represent different spatial or temporal phases of NO regulation rather than contradictory mechanisms. The most defensible interpretation is therefore that Si modifies the operating range and recovery dynamics of hormonal, Ca^2+^, NO, and ROS signaling networks. Direct evidence that Si is perceived by a specific receptor, generates distinct Ca^2+^ signatures, activates defined kinase cascades, or directly controls transcription-factor activity remains limited.

The second major mechanistic insight is that Si-associated stress mitigation extends beyond the plant and involves changes at the soil–root–microbiome interface. Si application can modify rhizosphere pH, nutrient availability, root metabolic output, and the quantity or composition of root exudates, thereby altering the chemical environment in which microbial communities assemble. These changes have been associated with the enrichment of microbial taxa and functional genes involved in nutrient mobilization, Na^+^ extrusion, K^+^ uptake, osmotic adaptation, biofilm formation, redox protection, and pathogen suppression. In turn, recruited microorganisms may influence plant metabolism and signaling through nutrient transformation, organic acids, osmolytes, phytohormone-related compounds, and other microbial metabolites. The soybean system involving SiO_2_ nanoparticles, 5-aminovaleric acid, Sphingomonas recruitment, and a synthetic microbial community provides an important example of a mechanistically resolved sequence in which Si-containing treatment changes root metabolic output, the altered metabolite environment recruits functional microorganisms, and microbial activity subsequently reinforces plant ionic, osmotic, and redox homeostasis. However, most other studies remain based on community profiling, functional prediction, or correlation analysis and do not yet establish that microbiome changes are necessary or sufficient for the observed plant phenotype.

These findings support a context-dependent inside–outside model of Si action. In this model, Si uptake and spatial deposition first modify structural, ionic, water, and redox conditions within the plant. These changes alter the physiological context in which hormonal and second-messenger networks operate and may also reshape root metabolic output. Modified exudates and rhizosphere conditions influence microbial recruitment and functional activity, while microbial metabolites and nutrient transformations feedback to plant ionic balance, antioxidant regulation, osmotic adjustment, and signaling. The resulting response is therefore better understood as a coupled feedback system than as a linear pathway or a fixed “Si–plant–microbe alliance.” The relative contribution of each component depends on crop genotype, Si-accumulation capacity, developmental stage, tissue, salinity regime, soil properties, microbial background, Si formulation, dose, and application route.

Overall, Si should be viewed as a context-dependent systems modulator rather than as a universally established central regulator of plant salt tolerance. Its principal contribution may be to buffer damaging physicochemical conditions, preserve the responsiveness of endogenous stress-signaling networks, and modify rhizosphere metabolite–microbe interactions in ways that favor recovery of plant homeostasis. This framework reconciles positive, neutral, and adverse responses by recognizing that Si effectiveness depends on whether the applied treatment matches the biological limitations of the plant and the environmental conditions of the soil–plant system. Future mechanistic validation should therefore focus on resolving the sequence linking Si availability, cellular homeostasis, signaling dynamics, root metabolic output, microbial recruitment, and agronomically meaningful stress protection.

### 7.2 Future research directions: from mechanistic resolution to microbiome engineering and precision agriculture

Future Si research should move beyond single-endpoint experiments toward temporally and spatially resolved mechanistic analysis. Integrated multi-omics approaches combining transcriptomics, proteomics, phosphoproteomics, metabolomics, ionomics, epigenomics, and microbiome profiling should be applied to the same biological samples and linked with physiological phenotyping. Time-series designs should distinguish rapid signaling events from later metabolic acclimation, whereas single-cell and spatial approaches could resolve Si-associated responses in specific root, vascular, guard, and mesophyll cell populations. Such studies should include salt-only, Si-only, and salt-plus-Si treatments together with appropriate pH, counter-ion, and formulation controls. Integration with mutants, genome editing, live-cell reporters, and pathway-specific perturbations will be necessary to distinguish causal Si-responsive mechanisms from secondary correlations. Recent studies integrating physiological, metabolomic, and microbiome data provide an initial foundation (Chen et al., 2025), but comprehensive analyses that connect signaling, protein activity, metabolism, ion transport, microbial dynamics, and yield remain scarce.

A second priority is the transition from descriptive microbiome profiling to Si–microbiome engineering. Future studies should identify microorganisms that consistently respond to Si across saline soils, determine the metabolites and root-exudate signals responsible for their recruitment, and test whether these microorganisms are necessary and sufficient for Si-associated stress alleviation. Microbiome depletion, isolate re-inoculation, sterile-substrate experiments, synthetic-community reconstruction, and field transplantation will be particularly important for establishing causality. Recent evidence that SiNPs can promote 5-aminovaleric-acid-mediated recruitment of Sphingomonas, that Si fertilization can alter metabolite–microbe coupling and rhizosphere N cycling, and that Si quantum dots can reshape microbial functions under drought illustrates the potential of this approach (Chen et al., 2026; Meng et al., 2026; Qin et al., 2026). However, the stability, host specificity, ecological persistence, and field reproducibility of engineered Si-responsive consortia remain unresolved. Next-generation Si–microbe formulations should therefore be designed according to crop genotype, Si-accumulation capacity, soil type, salinity intensity, Si source, and microbial compatibility rather than as universally applicable inoculants.

Artificial intelligence (AI) may provide a bridge between mechanistic knowledge and field-level Si management. Machine-learning models could integrate soil electrical conductivity, plant-available Si, pH, texture, irrigation-water quality, weather, crop genotype, Lsi-transporter characteristics, tissue Si status, canopy temperature, spectral indices, chlorophyll fluorescence, and yield responses to predict where and when Si application is likely to be effective. Remote sensing, uncrewed aerial vehicle (UAV) imaging, proximal soil sensors, and automated phenotyping could provide repeated measurements of salinity and crop stress, while interpretable models could estimate the optimal Si source, dose, timing, and application route. Machine learning and satellite- or UAV-derived variables have already been used to map within-field soil salinity, demonstrating the technical basis for spatially explicit management, although comparable models for predicting crop responses to Si have not yet been developed (Chaaou et al., 2025; Li et al., 2025). Future AI models should report uncertainty, undergo external validation across soils, seasons, and genotypes, and avoid extrapolation beyond the treatment conditions represented in their training data.

These developments could support precision Si management in which treatments are matched to spatial and temporal variation rather than applied uniformly. Field-scale salinity maps, plant-available soil Si, crop genotype, weather, and real-time crop phenotyping could be integrated to delineate management zones and optimize Si source, dose, timing, and application route. Closed-loop decision systems could subsequently update recommendations according to crop responses during the growing season. Because Si responses may become neutral or adverse outside an effective treatment window, precision management should prioritize the minimum effective dose rather than maximizing Si input. The resulting decision models should be validated across multiple locations, seasons, crop genotypes, and salinity environments.

To support the development of transferable Si-management strategies, future research should also prioritize data standardization and model transparency. Harmonized metadata, standardized reporting of Si source, formulation, dose, particle properties, application route, soil conditions, and salinity regime will be essential for comparing experiments and integrating datasets. Open multi-omics, microbiome, phenotyping, remote-sensing, and field-performance datasets should be developed using interoperable formats, while AI-assisted decision models should report uncertainty, feature importance, training boundaries, and external-validation performance. Ecological and regulatory validation should follow the formulation-specific and long-term assessment criteria outlined in Section 6.3. The ultimate goal is an evidence-based framework that connects mechanistic understanding, microbiome engineering, digital monitoring, and site-specific Si management.
